# A viral metagenomic approach on a non-metagenomic experiment: Mining next generation sequencing datasets from pig DNA identified several porcine parvoviruses for a retrospective evaluation of viral infections

**DOI:** 10.1371/journal.pone.0179462

**Published:** 2017-06-29

**Authors:** Samuele Bovo, Gianluca Mazzoni, Anisa Ribani, Valerio Joe Utzeri, Francesca Bertolini, Giuseppina Schiavo, Luca Fontanesi

**Affiliations:** 1 Department of Agricultural and Food Sciences (DISTAL), Division of Animal Sciences, University of Bologna, Bologna, Italy; 2 Department of Biological, Geological, and Environmental Sciences (BiGeA), Biocomputing Group, University of Bologna, Bologna, Italy; 3 Department of Veterinary Clinical and Animal Sciences, University of Copenhagen, Copenhagen, Denmark; 4 Department of Animal Science, Iowa State University, Iowa, United States of America; Oklahoma State University, UNITED STATES

## Abstract

Shot-gun next generation sequencing (NGS) on whole DNA extracted from specimens collected from mammals often produces reads that are not mapped (i.e. unmapped reads) on the host reference genome and that are usually discarded as by-products of the experiments. In this study, we mined Ion Torrent reads obtained by sequencing DNA isolated from archived blood samples collected from 100 performance tested Italian Large White pigs. Two reduced representation libraries were prepared from two DNA pools constructed each from 50 equimolar DNA samples. Bioinformatic analyses were carried out to mine unmapped reads on the reference pig genome that were obtained from the two NGS datasets. *In silico* analyses included read mapping and sequence assembly approaches for a viral metagenomic analysis using the NCBI Viral Genome Resource. Our approach identified sequences matching several viruses of the *Parvoviridae* family: porcine parvovirus 2 (PPV2), PPV4, PPV5 and PPV6 and porcine bocavirus 1-H18 isolate (PBoV1-H18). The presence of these viruses was confirmed by PCR and Sanger sequencing of individual DNA samples. PPV2, PPV4, PPV5, PPV6 and PBoV1-H18 were all identified in samples collected in 1998–2007, 1998–2000, 1997–2000, 1998–2004 and 2003, respectively. For most of these viruses (PPV4, PPV5, PPV6 and PBoV1-H18) previous studies reported their first occurrence much later (from 5 to more than 10 years) than our identification period and in different geographic areas. Our study provided a retrospective evaluation of apparently asymptomatic parvovirus infected pigs providing information that could be important to define occurrence and prevalence of different parvoviruses in South Europe. This study demonstrated the potential of mining NGS datasets non-originally derived by metagenomics experiments for viral metagenomics analyses in a livestock species.

## Introduction

Next generation sequencing (NGS) technologies have largely increased dimensionality of DNA sequencing projects for many different applications in all fields of biology, including the possibility to perform metagenomic studies, leading to a tremendous explosion of data that will continue increasing trend in the future [1].

Metagenomics, defined as the sequencing of all nucleic acids present in a sample despite its origin (e.g. environmental, specimen-derived), can explore complex microbial communities, including viral components, in a culture- and sequence-independent manner, overcoming the limits of traditional detection techniques (e.g. [2–4]). Viral metagenomics has been increasingly used in clinical virology to identify new pathogenic viruses or to characterize the complexity of pathogenic states in humans and livestock (e.g. [5–7]).

One of the most relevant challenges of viral metagenomics derives by the fact that viral sequences are usually present at a very low proportion in the analyzed specimens compared to the host DNA sequences [8]. The number of DNA sequences obtained from a specific virus in a sample is correlated to the viral load in the samples under investigation [9]. Therefore, common viral metagenomic approaches include viral particles or viral nucleic acid enrichment steps or other analytical procedures that reduce or remove non-viral DNA [2].

Metagenomic sequence data are generally analyzed by applying bioinformatic pipelines that can be divided into two main classes: 1) sequence assembly approaches and 2) read mapping approaches. Both procedures are characterized by two common steps: i) a pre-processing phase and ii) the filtering of reads against the host genome to remove the remaining host DNA sequences (i.e. host subtraction procedure; [10]).

The sequence assembly procedure implies, in a first step, the assembly of reads into longer contiguous sequences (contigs). This is not a trivial procedure, due to the uneven abundance (and coverage) of viral species as well as to other technical issues (e.g. sample processing and library preparation) that produce a limited read overlap required by the assembler. Up to date, several tools have been tested for assembling metagenomic reads (e.g. [9–12]). Moreover, specific assemblers for metagenomic data have been developed (such as MetaVelvet) and tested against viral metagenomic datasets [10,12]. In a subsequent step, contigs are then used for homology searches against sequence databases. A first BLASTN search is usually performed against the NCBI nt/nr database (looking for a virus sequence as best hit) while for contigs not assigned at DNA levels search in protein databases using translated nucleotide sequences can be performed [12]. To facilitate virome studies from NGS data, a few sequence assembly approaches have been designed. For example, a web server implementing a *de novo* assembly pipeline from NGS data (VirFind) has been recently proposed for studies in plant virology and tested also in insect virology [13].

Read mapping approaches are less computationally demanding than sequence assembly procedures, so resulting well suited for diagnostics or as a first filter before more extensive analyses are performed [8]. In read mapping approaches, reads are mapped on a reference sequence dataset from an *ad hoc* built database (e.g. [14]).

Metagenomics has been used as a tool in veterinary medicine to discover new viruses or to disclose complex viral infections in livestock species (e.g. [15]). Several applications of viral metagenomics have also targeted the pig for different objectives. For example, viral metagenomics studies have described the pig fecal virome [16], have identified a novel porcine boca-like virus [17], have shown that domestic pigs might be potential reservoirs for Ndumu virus [18], have identified porcine parvoviruses in complex infections [19], and have shown the usefulness of archived specimens in detecting parvovirus infections [20], among several other important discoveries that have contributed to epidemiological evaluations of emerging virus diseases in this livestock species.

In general, all these experiments have been designed according to the usual viral metagenomic approaches that enriched viral-derived nucleic acids and mined sequencing data following standardized and classic approaches optimized for these purposes. However, the large amount of NGS data that has been already obtained from the pig (and the increasing amount of generated sequences that is expected in the future) may open new possibilities to apply viral metagenomics to data produced by experiments designed from many other purposes and that did not have as first objective the identification of any viral sequences.

In this study, we identified viral sequences in pig specimens by mining NGS data that were produced with the Ion Torrent PGM sequencer using two reduced representation libraries (RRL) obtained from pooled DNA of many different pigs [21]. Data were from an experiment that was not originally planned for a metagenomic study. Our metagenomic sequence data mining strategy combined different approaches that i) used as main viral sequence reference dataset the NCBI Viral Genomes Resource [22] and ii) considered unmapped reads on the reference pig genome as a potential source of sequences of viral origin [23].

## Materials and methods

### Datasets

All animals used in this study were kept according to Italian and European legislation for pig production and all procedures described were in compliance with national and European Union regulations for animal care and slaughtering. All animals were part of the routine Italian pig breeding programme and were slaughtered in a commercial authorized abattoir following standard procedures. All animals were not raised or sampled for the purpose of this study. As no treatment was given to any animals, no ethics approval was needed according to the rules of the animal research ethics committee of the University of Bologna based on the Italian legislation, as reported in the “DECRETO LEGISLATIVO 4 marzo 2014, n. 26”.

Next generation sequencing data were from a previous study [21]. The investigated pigs were from the Italian Large White breed. Briefly, two DNA pools (LibP and LibN) were constituted including DNA from 50 performance tested pigs each. Performance tested pigs have been described elsewhere [24–26]. DNA was extracted from lyophilized blood of all pigs individually, using the Wizard® Genomic DNA Purification kit (Promega Corporation, Madison, WI, USA). Then, extracted DNA was quantified and pooled at equimolar concentration to constitute the two DNA pools from which two RRLs were constructed and next generation sequencing was carried out using the Ion Torrent PGM (Life Technologies, Carlsbad, CA, USA) following the procedure described in [21].

Obtained reads were first filtered and trimmed using the Ion Torrent suite v.2.2 (Life Technologies). Data were then inspected with FastQC v.0.11.22 available at http://www.bioinformatics.bbsrc.ac.uk/projects/fastqc/. Then reads were trimmed and filtered using PRINSEQ lite v.0.20.4 [27] as follow: i) trimming at the 3’-end up to 140 bp, ii) trimming of the 5’-end and 3’-end for poly A/T sequences > 5, iii) trimming the 5’-end and 3’-end up to reach a base with a quality score > 20, iv) exclusion of reads having average quality < 20, and (v) exclusion of reads shorter than 20bp. Sequence data have been submitted to the European Nucleotide Archive database (EMBL, http://www.ebi.ac.uk/ena/) and are indexed with the accession number PRJEB15234. The use of this experimental design is not specific for the subsequent data mining approach.

### Databases

Bioinformatic analyses relied on different resources: i) the pig reference genome sequence, version Sscrofa10.2 [28], available at the Ensembl database (release 85, July 2016); ii) the NCBI Viral Genomes Resource (December 2016, http://www.ncbi.nlm.nih.gov/genome/viruses/), a collection of viral (and viroid) species represented by a reference sequence [22], iii) the NCBI nucleotide collection (nr/nt), a collection of sequences from different resources; and iv) the NCBI GSS database (dbGSS; release 130101), a collection of unannotated short single-read, primarily genomic sequences from GenBank (http://www.ncbi.nlm.nih.gov/genbank/dbgss/).

### *In silico* detection of virus sequences

**Fig 1:**
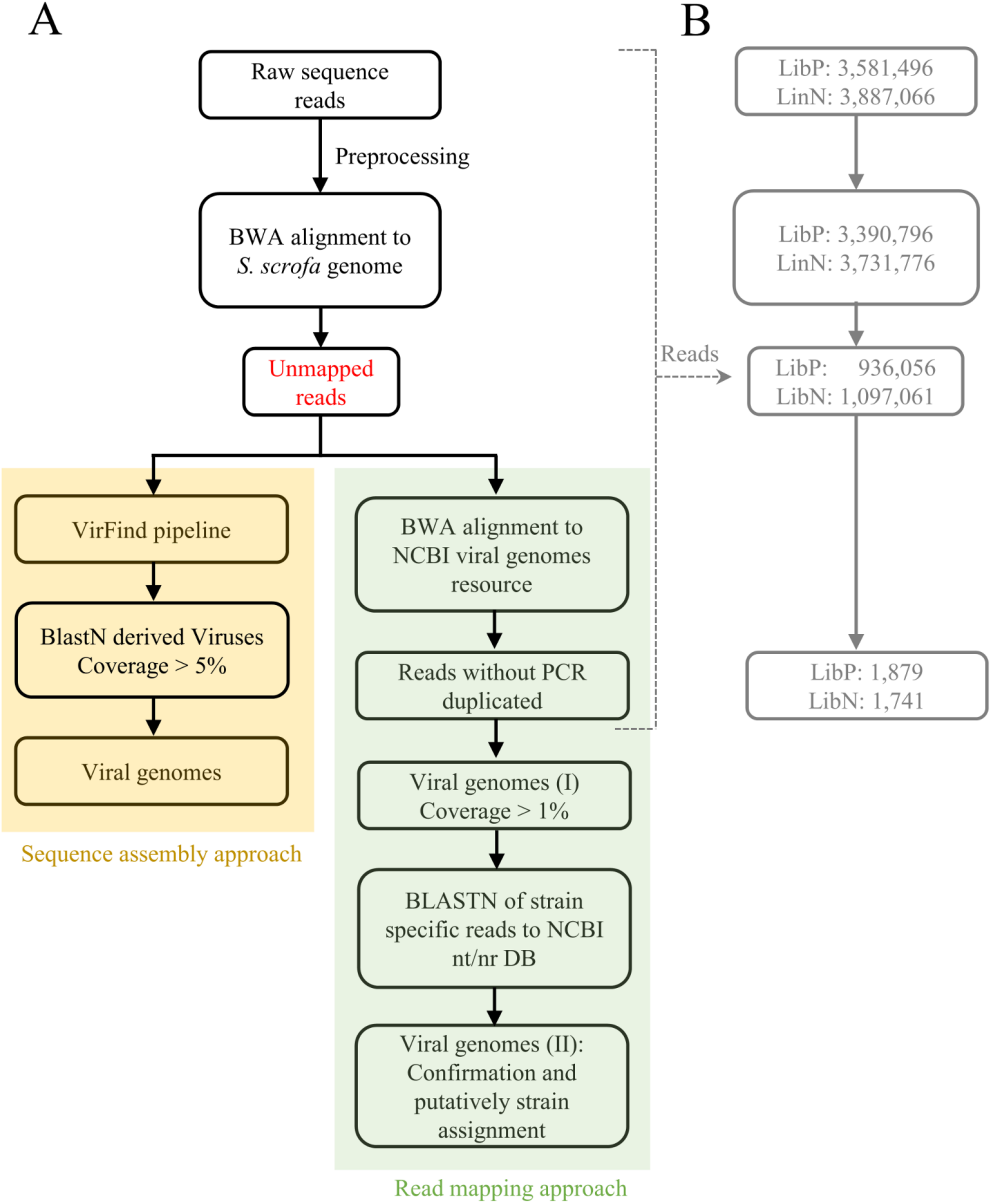
Flowchart of the bioinformatic analyses for identification of viral sequences from unmapped reads by using read mapping and sequence assembly approaches. a) Steps adopted in the virus discovery: after the preprocessing phase, reads were analyzed by using both a sequence assembly approach (yellow) and a read mapping approach (green). b) The flowchart proposed in box “a” is presented highlighting the number of reads obtained in each step of the analysis.

Virome characterization was carried out adopting read mapping and sequence assembly approaches as described below. For clarity, the flowchart of the bioinformatics pipeline is presented in Fig 1.

#### Read mapping approach

Trimmed and filtered reads were aligned by using BWA v.0.7.7 [29] on the Sscrofa10.2 pig genome. Unmapped reads were extracted and used for virome characterization. The presence of viral sequences was tested by using a read mapping approach. Unmapped reads were realigned, by using BWA, on the NCBI Viral Genomes Resource. Duplicate reads were first removed by using Picard v. 1.107 (http://picard.sourceforge.net). To test for the presence of viral genomes, we computed for each virus the coverage (number of bases covered by reads over the length of the genome), expressed as percentage. As each dataset originally showed a coverage of the reference pig genome of 5%, to accept the presence of a viral genome we set this threshold as limit of detection.

To refine the results, for a more specific taxonomic classification, reads mapped on a particular viral genome were analysed using BLASTN (http://www.ncbi.nlm.nih.gov/BLAST/) against the NCBI nucleotide collection (nr/nt). For each read all hits with an E-value < 0.0001 and showing the same statistics of the first hit were collected. The hit having the highest coverage was considered the closest viral strain or isolate.

#### Sequence assembly approach

VirFind web-service [13] was used for the sequence assembly approach. Unmapped reads were analyzed setting BLASTN E-value to 0.0001 and setting a coverage of 1% as limit of detection.

### PCR and Sanger sequencing analyses

The presence/absence of the *in silico* detected viruses was verified by PCR on DNA pools and on individual pig DNA samples. Primer pairs were designed with Primer3 v.0.4.0 (http://primer3.ut.ee/; Table 1). Primers were selected in the most conserved regions within virus species, identified using multiple sequence alignments (see notes to Table 1) obtained with Clustal Omega (http://www.ebi.ac.uk/Tools/msa/clustalo/; [30]).

**Table 1:** PCR primers and PCR conditions of the amplified fragments of the detected viruses.

Primer pair name/Virus ^a^	Primers (5’-3’)	PCR conditions ^b^	Expected Amplified region (bp)	Use
PPV2	Forward: CAGCAGACTGGCGATTTATT;Reverse: TTTGACGTAACCACCAGGAT	54/1.5	397	PCR/Sequencing
PPV4	Forward: TGGTTTTCCTGAGACTCCTG; Reverse: GTCGGCATTCTGTATTGTCC	58/1.5	250	PCR/Sequencing
PPV5	Forward: AAGGGGAAATTGGTGAAAAG; Reverse: TATATGGCGCCCAAATGTAT	54/1.5	351	PCR/Sequencing
PPV6	Forward: GACCTTCTGGACGGGTATTT; Reverse: TCAAGCCCTCTACACCAAAG	58/1.5	383	PCR/Sequencing
PBoV1-H18_317-616nt	Forward: GGTGAGTAACCATGCCTCTG; Reverse: GCGGTTTCAGCAAATATAGC	58/1.5	270	PCR/Sequencing
PBoV1-H18_1-316nt_317-616nt	Forward: GCACTCGCAGAAAGACTGTT; Reverse: CTTTCCGCATTCCTCTCTTT	58/1.5	226	PCR/Sequencing
PCV2_flank	Forward: AAGAATGGAAGAAGCGGACC; Reverse: CAAGGCTACCACAGTCACAA	59/1.5	429	PCR
PCV2_ovlp	Forward: CATTCAATGCAAGCGGTGTC; Reverse: CAAGGCTACCACAGTCACAA	59/1.5	263	PCR

^a^ Primers were built checking for conserved regions (if present) among the reference sequence and the different strains identified by using the refinement procedure adopted in the read mapping method. The following strains were used: PPV2—GenBank accession numbers: KP245947, GU938300, GU938301, KP765690, KC701309 and JX101461; PPV4—GenBank accession numbers: GQ387499 and GQ387500; PPV5—GenBank accession umbers: JX896319, JX896320 and JX896321; PPV6—GenBank accession: KF999682, KF999683, KF999684 and KF999685. Primers PBoV1-H18_317-616nt and PBoV1-H18_1-316nt_317-616nt were built based on the assembled sequence obtained by VirFind and on the reference sequence HQ291308. Primers PCV2_flank and PCV2_ovlp were built based on the assembled sequence obtained by VirFind and on the reference sequences KM259933 (truncated genome) and AY424401 (full genome adopted as reference).

^b^ Annealing temperature (°C) / [MgCl_2_].

PCR was carried out twice using a 2720 thermal cycler (Life Technologies, Carlsbad, CA, USA) in a 20 μL reaction volume containing ~50 ng of DNA, 1 U DNA Taq DNA polymerase (Kapa Biosystems, Wilmington, MA 01887, USA), 1X PCR buffer containing MgCl_2_, 2.5 mM dNTPs and 10 pmol of each primer. PCR cycle was as follow: 5 min at 95°C; 35 amplification cycles of 30 s at 95°C, 30 s at the specific annealing temperature for each primer pair (Table 1), 30 s at 72°C; 5 min at 72°C. Amplified DNA fragments were electrophoresed on 2.5% agarose gels and visualized with 1X GelRed Nucleic Acid Gel Stain (Biotium Inc., Hayward, CA, USA).

PCR fragments were sequenced using the BrightDye Terminator BrightDye® Terminator Cycle Sequencing Kit (Nimagen). Samples were loaded on an ABI3100 Genetic Avant Analyzer sequencer (Applied Biosystems, Carlsbad, CA, USA). Sanger sequence data were confirmed by BLASTN analyses.

## Results

### Preprocessing of NGS datasets

Ion Torrent sequencing of the two RRLs produced a total of 3,581,496 and 3,887,066 reads for the LibP and LibN, respectively ([21]; Table 2). A total of 936,056 and 1,097,061 reads (27.6% and 28.2% of the whole reads generated from LibP and LibN) did not map to the Sscrofa10.2 pig reference genome. These reads were classified as unmapped reads and used for the subsequent analyses.

**Table 2:** Summary of the Ion Torrent reads utilized for the detection of viral genomes. The number of reads are reported for the LibP and LibN DNA pools.

Information ^a^	LibP	LibN
Sequenced reads	3,581,496	3,887,066
Reads after preprocessing	3,390,796	3,731,776
Pig–Unmapped	936,056	1,097,061
Virus	9,926	11,752
Virus–no duplicates	1,879	1,741

^a^ “Pig–Unmapped” refers to reads unmapped on the *S*. *scrofa* nuclear genome; “Virus” refers to reads unmapped on the *S*. *scrofa* reference genome and mapping on viral genomes; “Virus–no duplicates” is the same of “Virus”, but after removing PCR duplicates.

### Read mapping detection of viral sequences

Re-alignment of unmapped reads against the NCBI Viral Genomes Resource, followed by the pruning of PCR duplicates (Fig 1), produced 1,879 and 1,741 reads (Table 2). These values corresponded to 0.20% and 0.19% of the unmapped fraction of LibP and LibN, respectively.

The largest number of reads from both libraries (with a total of 399 reads) matched the porcine endogenous retrovirus E (PERV-E) genome. These reads might derive from integrated retroviruses into the porcine genome that were not filtered out by the *in silico* analyses (considering the homology level we set). If we exclude PERV-E matched reads, other seven and six different groups of viral genome sequences were identified among LibP and LibN generated reads, respectively (S1 Table). Most of these detected viral sequences in both libraries matched sequences of porcine *parvovirus* 2 (PPV2), PPV4, PPV5 and PPV6, for a total of 260 reads. PPV2 had the most covered genome (89.73%) in the LibP with a total of 164 reads and 9 produced contigs. In LibN, the largest number of reads (no. = 48) matched PPV4 genome with a coverage of 8.70%.

Other reads matched sequences of viruses that have not been previously associated or detected in pigs: Shamonda virus (15 reads, matching the small segment of the negative-stranded tripartite RNA genome), *Malvastrum* leaf curl Philippines betasatellite (nine reads) and *Glypta fumiferanae* ichnovirus (28 reads).

To confirm or filter out false positive matches obtained with the read mapping approach, the procedure was refined using BLASTN against the NCBI nucleotide collection (nr/nt). For each read all hits with an E-value < 0.0001 and showing the same statistics of the first hit were collected. Then, for each hit, coverage was computed to confirm and identify a possible viral strain from which sequences were putatively originated or were closely related. Hits having the highest coverage on a specific viral strain sequence entry (or group of entries) were assumed to putatively identify that specific viral strain or isolate. It should however be pointed out that, according to the pooling strategy of our experiment, it is possible that more than one strain for each detected virus could be present among these reads. This BLASTN refining step might identify the most frequent strain (and/or the closest putative strain) that provided the largest number of reads. Information on the first three entries ranked by BLASTN for each detected viral genome identified in the preliminary step of the read mapping approach is reported in S2 Table. Results of this BLASTN refining analysis for the putative viral sequences detected in the LibP and LipN datasets were the following (S3 Table): i) PERV-E sequences were confirmed as PERV-E only as third hits, further supporting that the identified matches were from integrated retrovirus sequences in the porcine genome (the first two hits were from pig DNA sequences); ii) PPV2 sequences matched with higher coverage different strains than that used as reference by the NCBI Viral Genome Resource; iii) PPV4, PPV5 and PPV6 viral sequences were confirmed to match the same strains used as reference by the NCBI Viral Genome Resource; iv) sequences that were supposed from *Malvastrum* leaf curl Philippines betasatellite, Shamonda virus and *Glypta fumiferanae* ichnovirus actually were not confirmed to derive from these viruses as they matched pig or human genomic sequences (suggesting that they originated from the host genome and not from contaminating viruses of the investigated specimens).

### Sequence assembly detection of viral genomes

The sequence assembly approach we used was based on VirFind web-service [13] for the detection of virus sequences (Fig 1). To overcome limits of the bioinformatic pipeline and to facilitate sequence mining, we analyzed only nuclear unmapped reads obtained as described above. Twenty-two and six contigs from LibP and LibN datasets aligned 17 and six viral genome entries, respectively (four were in common between the two datasets; S3 Table).

VirFind assembled 10 contigs (seven from LibP and three from LibN) that aligned with seven different retrovirus sequences (eight contigs from PERV sequences and two from Moloney Murine Leukemia Virus), probably derived by different integrating events in the porcine genome that remained in the unmapped read fraction. Other 11 contigs (all from the LibP dataset) aligned five PPV2 sequences from different strains (six contigs mapped on the same strain JH13 genome that was covered for a total of ~39% of its genome size). The longest PPV2 contig (577 nt) mapped on the strain YH14 genome (~10% of coverage).

Contigs matched with PPV5 and PPV6 genomes on both LibP and LibN datasets. Two PPV5 contigs were generated from LibP and one was obtained from LibN, all matching non-coding regions 5’ to the non-structural protein 1 (NS1) gene. A PPV6 contig obtained from LibN matched a 5’ flanking region to the NS1 gene. Another contig constructed for this virus from LibP was quite puzzling as it seems to have been generated from a circularized genome. BLASTN analysis identified two matched regions at its extreme ends to the most similar GenBank entry (KR709268), regions that were identical or almost identical to corresponding sequences at the beginning and at the end of the reported virus genome (nucleotides 1–112 and 6148–6103 of KR709268 matched with nucleotides 286–397 and 24–70 of contig1 with 100% and 98% identical matches, respectively). As parvoviruses have linear genomes this result could be also obtained by a chimeric sequence or could be derived by an artifact of the assembly procedure. Further studies are needed to clarify this question.

A match with the PPV4 genome (in the 5’ flanking region of the NS1 gene) was identified only in the LibN dataset. In the LibP dataset, four reads aligned to the PPV4 genome, producing three contigs. A long contig (from 12–431 nt) was not built (even if the presence of PPV4 sequences was evident from visually inspected alignments) since there were no overlapping reads in the region around the 217 nt, explaining only a genome coverage <1% that was not reported in S3 Table (data not shown).

VirFind contigs matched genome sequences of two other viruses not previously detected with the read mapping approach: porcine *bocavirus* 1-H18 isolate (PBoV1-H18; in LibP) with the longest assembled contig of this approach (616 bp) and porcine *circovirus* 2 (PCV2), strain ZJ-R, truncated genome sequence (in LibP and LibN).

Identity of the assembled contigs with the matched virus genome regions ranged from about 95 to 100% (S3 Table). As most of these contigs were constructed from more than one infected animal (considering the DNA pool approach and the individual validation analyses described below) they might not be considered as derived from a single isolate as this question was not addressed in our study. All generated contigs are available in the S1 File.

### PCR validation of *in silico* detected viruses

**Fig 2:**
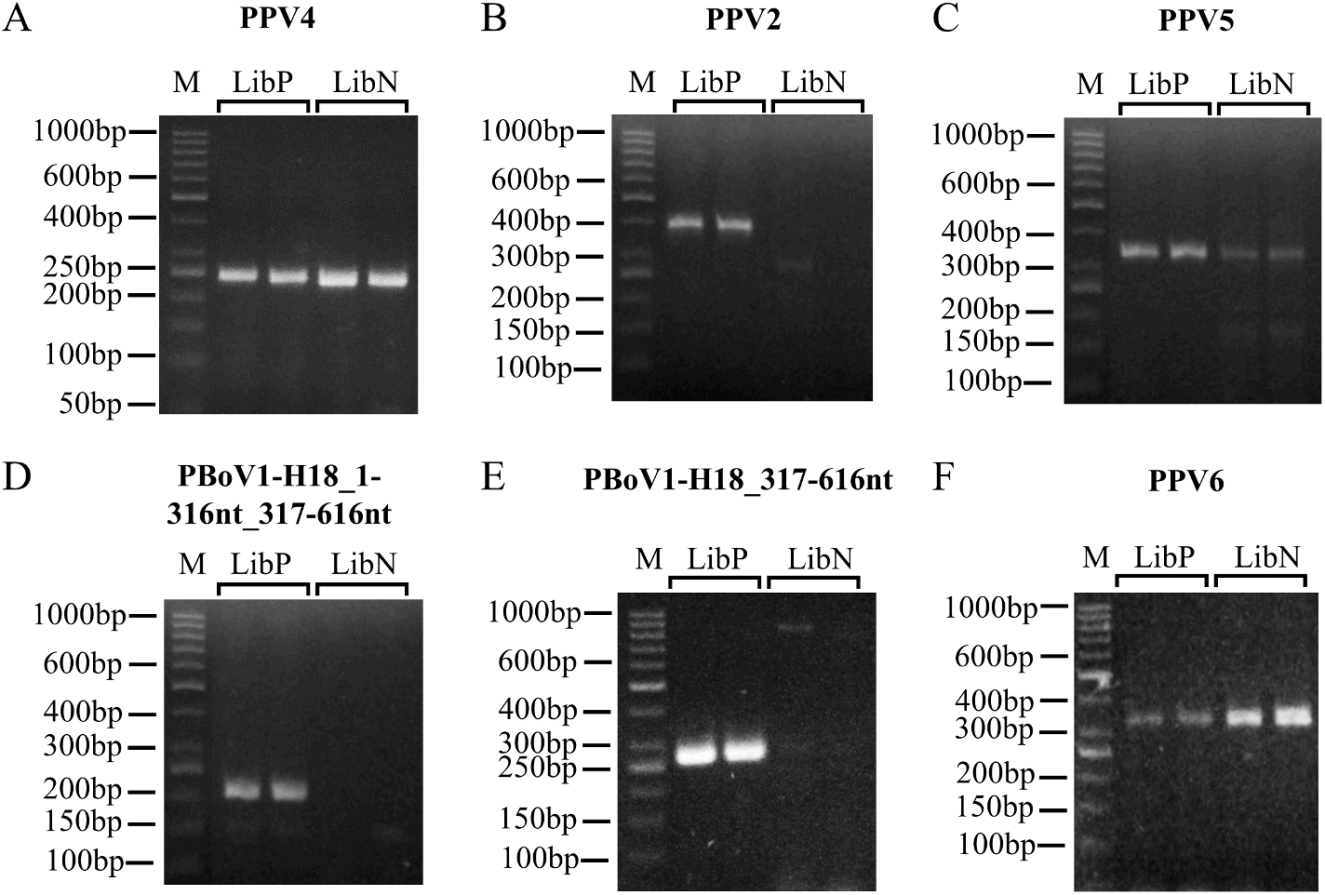
PCR validation of the *in silico* detected porcine viruses. Boxes are named after the primer pairs utilized for the validation reported in Table 1. a) PPV4; b) PPV2; c) PPV5; d) PBoV1-H18_1-316nt_317-616nt; e) PBoV1-H18_317-616nt and f) PPV6. Each box presents the following columns: “M”—ladders molecular size markers; “LibP”—amplification products (in duplicate) in the LibP DNA pool; “LibN”—amplification products (in duplicate) in the LibN DNA pool.

A PCR primer pair was designed for each detected parvovirus (PPV2, PPV4, PPV5 and PPV6; Table 1) to validate the presence of these DNA fragments in the original DNA pools. PCR analyses confirmed the presence of PPV4, PPV5 and PPV6 DNA in both DNA pools (Fig 2A, Fig 2C and Fig 2F, respectively) whereas PPV2 DNA was detected only in the LibP DNA pool probably due to the very low load and diluted sequences of this virus in the animals of this DNA pool (Fig 2B; Table 3). Sanger sequencing of the obtained amplicons confirmed that the amplified fragments corresponded to what was expected (GenBank/EMBL accession numbers: LT622854 and LT622855 for PPV2; LT622856 for PPV4; LT622857 and LT622858 for PPV5; LT622859 and LT622860 for PPV6; LT622861).

**Table 3:** Summary of the viruses identified in the two next generation sequencing datasets (LibP and LibN). Identification was obtained by *in silico* analyses (with the read mapping and sequence assembly approaches) and on DNA samples from which libraries were generated by PCR analyses on DNA pools and on individual DNA samples.

Virus/Primer pair	DNA pools (*in silico*): rm/sa ^a^		DNA pools (PCR) ^b^		Individual DNA samples (PCR) ^c^	
	LibP	LibN	LibP	LibN	LibP	LibN
PPV2	+/+	+/-	+	-	5	0
PPV4	+/-	+/+	+	+	7	5
PPV5	+/+	+/+	+	+	4	3
PPV6	+/+	+/+	+	+	6	7
PBoV1-H18_1-316nt_317-616nt	-/+	-/-	+	-	1	NA^*^
PBoV1-H18_317-616nt	-/+	-/-	+	-	1	NA^*^

^a^ Identification of viral sequences by *in silico* analyses: rm = read mapping approach; sa = sequence assembly approach. “+” indicates presence of sequences; “-” indicates absence of sequences.

^b^ Identification of the presence of viral sequences by PCR analysis on DNA pools: “+” indicates presence of amplification (positive); “-” indicates absence of amplification (negative).

^c^ Identification of the presence of viral sequences by PCR analysis on individual DNA samples. The number of positive samples is reported out of 50 pigs for the two groups (LibP and LibN).

^*^ Not amplified.

Two PCR primer pairs were used to verify the presence of PBoV1-H18 DNA in the original DNA samples (S1 Fig). Only a region from nucleotides 317 to 616 (317-616nt) of a 616 bp long PBoV1-H18 contig (Contig_1–616) perfectly aligned with the viral reference genome, leaving ~50% of the remaining contig as unaligned (region 1-316nt). To exclude that the Contig_1–616 would have been derived by an artefact of the *de novo* assembly, we proved the real existence of the two parts of this contig as follows: the first primer pair was designed within the 317-616nt region, while the second pair was constituted by a forward primer placed in the 1-316nt region and a reverse primer selected in the 317-616nt region (PBoV1-H18_1-316nt_317-616nt primer pair; Table 1). The presence of the PBoV1-H18 DNA was confirmed in the LibP DNA pool by both primer pairs (Fig 2D and Fig 2E). Sanger sequencing of the two amplicons confirmed the expected DNA, including the amplification of the 1-316nt_317-616nt fragment (S1 Fig; GenBank/EMBL accession no. LT622861). No amplification was obtained for both PCR primer pairs when tested using LibN pool DNA, confirming the results of the sequence assembly bioinformatic analysis (Fig 2D and Fig 2E). Amplification of individual DNA samples confirmed the results of the DNA pool analyses (Table 3).

The design of a PCR validation strategy for PCV2 ZJ-R was adapted to the specific sequence composition of this strain. Normally, PCV2 has a single-stranded circular genome of 1766 nucleotides. The ZJ-R strain have a small circular genome sequence of 694 nucleotides (truncated genome), containing a 180 nucleotide non-viral insertion (with 99% homology with a swine derived sequence; GenBank accession number: HE214143; that also shows a high number of BLAST matches with P<0.0001 in the pig genome; data not shown), for which possible recombination with cellular protein-coding sequences was observed [31]. For this virus strain, a PCR primer pair (PCV2_flank; Table 1) was designed on the virus specific sequence (highly conserved in many different PCV2 strains; data not shown) that flanked the inserted sequence. A second PCR primer pair (PCV2_ovlp; Table 1) included a forward primer placed in the pig DNA homolog part and a reverse primer in the virus specific region. No amplification was obtained on both DNA pools (LibP and LibN). A retrospective further look at sequences of the putative PCV2 contig generated by VirFind was obtained by BLASTN analysis against the dbGSS resource, that identified an almost complete alignment (~95%) of this sequence with several porcine genomic sequences (S2 File). These additional results, taken together with the lack of PCR amplification, may indicate that the sequence assembly analysis generated a spurious sequence match attributed to the PCV2 ZJ-R strain due to the particularity of its genome sequence that includes a host-derived inserted sequence.

### Analysis of individual pig DNA samples

A retrospective analysis of the positively amplified DNA samples extracted from the performance tested pigs could provide interesting information from the occurrence, distribution and prevalence of virus infections and co-infections (S4 Table). The analysed pigs were performance tested in a period spanning 11 years (from 1996 to 2007) and coming from different Italian farms [21]. Considering the 100 individually PCR amplified pigs, 31% were infected by at least one virus. Among these pigs, 7 were infected by two different parvoviruses. The first detected parvovirus positive pig, infected with PPV5, was slaughtered in 1997. The other PPV5 positive pigs were slaughtered in 1998 (n. 2; from two different slaughtering batches), 1999 (n. 2; from two different slaughtering batches) and 2000 (n. 2; from the same slaughtering batch). One of these pigs was also positive for PPV4 whereas one of the PPV5 positive pig slaughtered in 1998 and one slaughtered in 1999 were also positive for PPV6. Two other pigs slaughtered in 1998 were positive for PPV6 in our study. The remaining pigs infected by PPV6 were slaughtered from 2000 to 2004 (S4 Table). PPV6 was the most frequent detected virus in our analysed individual pigs (13% of the analysed samples). PPV4 was the second most frequently detected parvovirus in the analysed pigs (12%) whereas PPV2 and *bocavirus* were the least frequent ones (5% and 1%, respectively). Two pigs were co-infected by PPV2 and PPV4. It is worth to mention that previous studies that first described several parvoviruses identified also in our study (i.e. PPV5, PPV6 and PBoV1-H18; i.e. [20, 32]) reported their occurrence in specimens isolated from pigs living in a period spanning from 2006 to 2010, later than the life span period of our archived samples.

## Discussion

The large amount of NGS data already available in most livestock species is opening new possibilities for mining these sequence datasets to extract additional information that was not the main target of the original experiments. DNA extraction procedures from tissue specimens not only isolate genomic DNA of the host organism but also of all other infecting microorganisms and viruses contained in the sampled tissues and cells. Therefore, NGS datasets generated from shot-gun sequencing approaches may contain reads that are derived from infecting organisms potentially transforming the original experiment in a metagenomic study even if not previously designed for this specific purpose [33]. One of the main challenges is however the application of appropriate bioinformatic steps that can extract relevant information from the large amount of generated sequences coming from the host organism.

To this aim, we first filtered generated reads by using the Sscrofa10.2 porcine reference genome [28] and considered for subsequent analyses only unmapped reads that might be enriched of sequences that could not be derived from the genomic DNA of the host organism. Unmapped reads have been also used in [23] to identify absent, incomplete or misassembled genomic sequences on the reference *Bos taurus* genome version and nematode sequences derived by a blood-borne parasite that was unexpectedly found to have infected the cattle whose DNA was used to construct the bovine reference genome. It is also clear from our study that most of the unmapped reads are derived from porcine genomic sequences not yet incorporated in the Sscrofa 10.2, considering the preliminary and incomplete assembly of the currently available porcine reference genome [34]. For example, several unmapped reads in our study matched PERV sequences that are retroviruses (whose sequences are included in the NCBI Viral Genomes Resource; [22]), known to pervade the *Sus scrofa* genome but that did not match (considering the used thresholds) any other sequence of the Sscrofa10.2 genome version (and for this reason were not filtered out by the preliminary mapping approach against the host pig genome). This is probably due to the fact that the reference pig genome does not contain all types of PERV sequences that however were integrated in the genome of the pigs that were used to construct sequenced RRLs. It is well known that different pig lines or breeds have different contents of these retrovirus sequences (e.g. [35–36]).

Despite these problems, using virus genome sequences available in the NCBI Viral Genomes Resource [22] to align unmapped reads, we identified viral sequences produced by several parvoviruses that originally infected the pigs that were used in the construction of the DNA pools. Our study and [23] demonstrated that it is possible to consider NGS datasets as source of metagenomics information. We could envisage that routine mining of NGS data against viral genome sequences might become a common method to discover novel viral infections and novel viruses in the future. One limit could be due to the absence of reference viral sequences for all viruses in the NCBI Viral Genomes Resource database that is used for sequence alignments, even if this resource is in rapid expansion [37–38]. That means that it could be also possible to obtain novel information by re-analysing NGS datasets against future updated versions of the reference viral database.

A few examples could be mentioned to evaluate the strength and limits of our viral metagenomic study that can be refined or adapted according to the features of the mined NGS datasets. PPV4 sequences were detected by the read mapping method in both LibP and LibN but was only confirmed with the sequence assembly approach in the LibN as in its dataset the small number of reads did not provide enough overlapping sequences to construct any contig. The presence of PPV4 sequences in LibN was however validated by *in vitro* analyses. That means that viral load, that in turn may affect read depth and coverage related to the genome of the infecting organisms (in particular, in an experiment that already has a low coverage and read depth related to the porcine reference genome; [21]) is very important to obtain a validation with the sequence assembly approach and relaxed thresholds might be needed in this specific case.

Another example was provided by PCV2 sequences that were detected with the sequence assembly approach but results were not confirmed by additional laboratory investigations and close evaluation of the bioinformatic analyses. PCR validation did not confirm the presence of PCV2 sequences that were *in silico* attributed to a peculiar strain (ZJ-R) whose genome includes a host-derived sequence [31]. This part of its genome, that was not possible to distinguish (just by looking at the raw bioinformatic outputs) from host derived sequences that remained among unmapped reads, was the source of this false positive result.

An example in which NGS reads might be more powerful than PCR validation could be provided by the detection of PPV2 sequences in the LibN dataset. The read mapping approach (that might perform better when read depth and genome coverage are very low) identified the presence of PPV2 sequences coming from this library (only 4 reads in the pool). PCR could not validate the presence of this (putative) very low virus load (eventually diluted in different animals) probably due to the sub-optimized PCR efficiency for this PPV2 amplified region. Low PCR efficiency was suggested to be the cause of partially validated results also in other parvovirus detection experiments (e.g. [20]).

As pigs from which DNA was extracted to construct DNA pools were slaughtered in a period from 1996 to 2007, our study retrospectively identified pigs that were infected by several parvoviruses. Subsequent analyses on individual DNA samples showed that several pigs were also co-infected by more than one virus. It is worth to mention that, according to available veterinary records, all investigated animals were considered healthy, without any apparent signs of infections. In addition, as all these pigs were part of a performance testing selection program [21], in which only veterinary inspected animals could be moved in a performance testing station, all animals were analysed following the routine laboratory tests needed at that time and were not considered positive for any pathogen under monitoring. Several other studies reported apparently asymptomatic infections from viruses of the *Parvoviridae* family in many different pig herds and countries with sometimes a very high prevalence of positive animals [39–41]. As our archived samples were from performance tested pigs, it could be interesting to use growth data of these animals to evaluate if infected animals, that were apparently asymptomatic, could show sign of subclinical infections (and that could be eventually considered as pre-clinical cases, considering also the quite low infection loads that they showed), by comparing their growth rates with contemporary pigs that were negative at the PCR tests we designed.

According to the year when investigated pigs were sampled, we could mention that PPV5-related viruses circulated in Italian pigs much earlier than the first detection of positive animals for PPV5 in the world. This virus was identified in US archived samples collected in 2006 [20], after its first identification in pigs that lived in 2013 [42]. In our study, PPV5-positive pigs were slaughtered in a period ranging from 1997 to 2000.

Again, we detected PPV6-related sequences in pigs that were slaughtered even in 1998 (and in a period spanning 1998–2004), more than 10 years before this virus was first recognized in China from specimens collected in 2010–2013 [32] and then identified in the United States and Mexico [19].

In our study, pigs that were infected by PPV2 were slaughtered in 1998, 2000, 2003 and 2007 whereas pigs that were positive for PPV4 sequences were slaughtered in 1998–2000. PPV4 was first discovered in archived samples collected in 2005 during high mortality outbreaks associated with porcine circovirus associated disease in North Carolina [43] and subsequently detected in tissues collected again in US in 1998 [20], matching the earliest detection we obtained in a few Italian samples. The first detection of the porcine bocavirus 1 isolate H18 (PBoV1-H18) was reported in specimens collected in several Chinese provinces in 2008–2009 [44]. This virus was then classified into the PBoV G1 group [45] and first detected in Sweden in pigs infected also with PCV2 [17]. In our study, only one Italian Large White pig slaughtered in 2003 was positive for PBoV1-H18 indicating that similar isolates circulated in South Europe a few years earlier than the first detection in China and North Europe.

Cadar *et al*. [46] suggested that PPV2, PPV3 (both probably originated in Romania) and PPV4 (probably originated in Croatia) have been present in European domestic pig populations at least since the 1920s, 1930s and 1980s, respectively. It could be important to analyze additional historic samples to evaluate when genetic diversity among the *Parvovinae* subfamily emerged. Moreover, phylogenetic analyses including our sequences will be useful to clarify the precise origin of the parvoviruses we detected in our investigation. However, contigs we obtained from the sequence assembly approach could be of limited value for this purpose as they provided short sequences mainly from non-coding regions of different parvoviruses. Isolation of full genome sequences from our historic samples might help to better address this question.

## Conclusions

This study demonstrated the potential of mining NGS datasets non-originally derived by metagenomic experiments for viral metagenomic analyses that might be useful to disclose new information and to clarify the origin of viruses infecting livestock species. We showed that interesting sequence information remains in the unmapped fraction of NSG datasets. This fraction can be explored for many other purposes than those derived by the experiments that generated these datasets.

Results we obtained made it possible to provide a retrospective analysis of putative asymptomatic viral infections of pigs raised in Italy. Using archived samples, we identified pigs that were infected by several viruses of the *Parvoviridae* family, indirectly demonstrating that their genetic differentiation might have occurred earlier and in different geographic regions than those previously reported, opening new questions and opportunities for virus epidemiological studies.

## Supporting information

S1 Table Viruses identified in the two libraries (LibP and LibN) by using the read mapping approach. (DOCX)

S2 Table Refinement of the read mapping approach obtained by BLASTN analysis. (DOCX)

S3 Table Viruses identified in the two libraries (LibP and LibN) by using the sequence assembly approach. (DOCX)

S4 Table List of pigs (identified by each line in the table) that were individually PCR amplified to detected the presence of different parvoviruses using primers reported in Table 1. (DOCX)

S1 Fig Sequence validation of the PBoV-H18 virus. (A) The forward primers of the PBoV1-H18_1-316nt_317-616nt and PBoV1-H18_317-616nt primer pairs are highlighted in green and red, respectively. Nucleotides of the Contig_1–616 aligned by using VirFind are in bold. “+” indicates a sequence identity between the portion of the Contig_1–616 not aligned by using VirFind and the DNA region sequenced by using primer pair PBoV1-H18_1-316nt_317-616nt (100% identity). Sequence identity between these 35nt and the PBoV1-H18 genome (GenBank: HQ291308) is equal to 20% indicating that the portion upstream the region recovered by VirFind could be (at least for these 35 bases), very dissimilar from the reference HQ291308. “*” indicates a sequence identity among the Contig_1–616, the reference and the two obtained Sanger sequences. (B) Sanger sequence of part of the Contig_1–616. The primer and the region aligned with VirFind are highlighted.
(TIF)

S1 File List of contigs generated for the LibP and LibN datasets with the sequence assembly approach. (DOC)

S2 File Alignment of the PCV2 contig with BLASTN matched porcine genomic sequences. (DOC)
