# Supplementary material for: A viral metagenomic approach on a non-metagenomic experiment: Mining next generation sequencing datasets from pig DNA identified several porcine parvoviruses for a retrospective evaluation of viral infections
Source: PLoS One. 2017 Jun 29;12(6):e0179462. doi: 10.1371/journal.pone.0179462 (PMC5491021; doi:10.1371/journal.pone.0179462)
Supplement: S1 Table — (DOCX) [file pone.0179462.s001.docx]

**S1 Table. Viruses identified in the two libraries (LibP and LibN) by using the read mapping approach.**

| **Library** | **RefSeq ^a^** | **GenBank ^b^** | **Name ^c^** | **Genome size ^d^** | **Covered bases ^e^** | **% of coverage ^f^** | **RD max ^g^** | **RD mean ^h^** | **Reads ^i^** | **Contigs ^j^** |
| --- | --- | --- | --- | --- | --- | --- | --- | --- | --- | --- |
| LibP |  |  |  |  |  |  |  |  |  |  |
|  | NC_025965 | KM926355 | PPV2 – BR/GO/ion_09_PPV-2/2011 | 5426 | 4869 | 89.73 | 11 | 3.67 | 164 | 9 |
|  | NC_003059 | AF356697 | PERV-E | 8072 | 6346 | 78.62 | 15 | 3.07 | 196 | 20 |
|  | NC_021929 | KF433066 | *Malvastrum* leaf curl Philippines betasatellite | 673 | 82 | 12.18 | 6 | 2.67 | 7 | 2 |
|  | NC_023020 | JX896321 | PPV5 – IA469 | 5805 | 576 | 9.92 | 3 | 1.43 | 8 | 4 |
|  | NC_023860 | KF999685 | PPV6 – TJ | 6148 | 601 | 9.78 | 4 | 1.28 | 8 | 5 |
|  | NC_018464 | HE795107 | Shamonda virus N and NSs genes | 927 | 72 | 7.77 | 12 | 10.07 | 12 | 1 |
|  | NC_008912 | AB289986 | *Glypta fumiferanae* ichnovirus segment C9 | 3141 | 199 | 6.34 | 22 | 6.16 | 28 | 3 |
|  | NC_014665 | GQ387499 | PPV4 – Clone_17 | 5905 | 369 | 6.25 | 3 | 1.40 | 5 | 3 |
| LibN |  |  |  |  |  |  |  |  |  |  |
|  | NC_003059 | AF356697 | PERV-E | 8072 | 6596 | 81.71 | 13 | 3.07 | 203 | 21 |
|  | NC_023020 | JX896321 | PPV5 – IA469 | 5805 | 691 | 11.90 | 3 | 1.50 | 10 | 5 |
|  | NC_023860 | KF999685 | PPV6 – TJ | 6148 | 594 | 9.66 | 7 | 2.45 | 13 | 2 |
|  | NC_014665 | GQ387499 | PPV4 – Clone_17 | 5905 | 514 | 8.70 | 26 | 9.56 | 48 | 2 |
|  | NC_025965 | KM926355 | PPV2 – BR/GO/ion_09_PPV-2/2011 | 5426 | 417 | 7.69 | 1 | 1.00 | 4 | 4 |
|  | NC_018464 | HE795107 | Shamonda virus | 927 | 64 | 6.90 | 3 | 2.88 | 3 | 1 |
|  | NC_021929 | KF433066 | *Malvastrum* leaf curl Philippines betasatellite | 673 | 44 | 6.54 | 2 | 1.50 | 2 | 1 |

^a^ Reference Sequence accession number of the matched virus genome

^b^ GenBank accession number of the matched sequence

^c^ Virus – Short name. Full name and description are available in the GenBank entry.

^d^ Genome size (in bp) of the RefSeq

^e^ Virus genome nucleotides covered by Ion Torrent reads.

^f^ Percentage of virus genome nucleotides covered by Ion Torrent reads.

^g^ Maximum Read Depth (RD).

^h^ Mean Read Depth (RD).

^i^ Number of mapped reads.

^j^ Number of sequenced continuous regions.
