# Supplementary material for: A viral metagenomic approach on a non-metagenomic experiment: Mining next generation sequencing datasets from pig DNA identified several porcine parvoviruses for a retrospective evaluation of viral infections
Source: PLoS One. 2017 Jun 29;12(6):e0179462. doi: 10.1371/journal.pone.0179462 (PMC5491021; doi:10.1371/journal.pone.0179462)
Supplement: S2 Table — (DOCX) [file pone.0179462.s002.docx]

**S2 Table. Refinement of the read mapping approach obtained by BLASTN analysis.** The first four BLASTN hits are reported together with the Reference NCBI sequence reported in S1 Table to define the corresponding sequences.

| **Genome^a^** | **LibP dataset*** | | | **LibN dataset*** | | |
| --- | --- | --- | --- | --- | --- | --- |
|  | **GenBank^b^** | **Name^c^** | **nt^d^** | **GenBank^b^** | **Name^c^** | **nt^d^** |
| KM926355 | 1. GU938300 2. GU938301 3. KP245947 4. KM926355 | 1. PPV2 – JH13 2. PPV2 – YH14 3. PPV2 – 215 4. PPV2 – BR/GO/ion_09_PPV-2/2011 | 1. 3433 2. 2472 3. 2430 4. 600 | 1. KP765690 2. GU938300 3. JX101461 4. KM926355 | 1. PPV2 2. PPV2 – JH13 3. PPV2 – 215 4. PPV2 – BR/GO/ion_09_PPV-2/2011 | 1. 249 2. 206 3. 134 4. 0 |
| AF356697 | 1. CU480790 2. CU861973 3. AF356697 4. AF356697 | 1. Pig DNA sequence – ChrX 2. Pig DNA sequence – ChrX 3. PERV-E 4. PERV-E | 1. 1554 2. 1491 3. 1377 4. 1377 | 1. CU480790 2. FP091239 3. CU861973 4. AF356697 | 1. Pig DNA sequence – ChrX 2. Pig DNA sequence – ChrX 3. Pig DNA sequence – ChrX 4. PERV-E | 1. 1795 2. 1748 3. 1487 4. 1345 |
| KF433066 | 1. FP245529 2. FP700183 3. CU463884 4. KF433066 | 1. Pig DNA sequence – ChrX 2. Pig DNA sequence – ChrX 3. Pig DNA sequence – Chr2 4. Malvastrum leaf curl Philippines betasatellite | 1. 130 2. 107 3. 97 4. 0 | 1. CR974434 2. FP102047 3. CP011903 4. KF433066 | 1. Pig DNA sequence – Chr7 2. Pig DNA sequence – ChrX 3. *Ovis canadensis* – Chr18 4. *Malvastrum* leaf curl Philippines betasatellite | 1. 106 2. 79 3. 57 4. 0 |
| JX896321 | 1. JX896321 2. JX896320 3. JX896319 4. JX896321 | 1. PPV5 – IA469 2. PPV5 – IA469 3. PPV5 – IN273 4. PPV5 – IA469 | 1. 479 2. 456 3. 358 4. 479 | 1. JX896320 2. JX896321 3. JX896322 4. JX896321 | 1. PPV5 – IA469 2. PPV5 – IA469 3. PPV5 – ND564 4. PPV5 – IA469 | 1. 410 2. 410 3. 335 4. 410 |
| KF999685 | 1. KF999685 2. KF999683 3. KF999684 4. KF999685 | 1. PPV6 – TJ 2. PPV6 – JS 3. PPV6 – SC 4. PPV6 – TJ | 1. 356 2. 253 3. 253 4. 356 | 1. KF999685 2. KF999684 3. KF999682 4. KF999685 | 1. PPV6 – TJ 2. PPV6 – JS 3. PPV6 – BJ2 4. PPV6 – TJ | 1. 548 2. 234 3. 217 4. 548 |
| HE795107 | 1. AL353644 2. FP236383 3. AL592188 4. HE795107 | 1. Pig DNA sequence – Chr22 2. Pig DNA sequence – Chr21 3. Human DNA sequence 4. Shamonda virus | 1. 296 2. 296 3. 296 4. 0 | 1. AL353644 2. FP236383 3. AL592188 4. HE795107 | 1. Pig DNA sequence – Chr22 2. Pig DNA sequence – Chr21 3. Human DNA sequence 4. Shamonda virus | 1. 376 2. 376 3. 376 4. 0 |
| GQ387499 | 1. GQ387499 2. JX896319 3. GQ387500 4. GQ387499 | 1. PPV4 – Clone_17 2. PPV5 – IN273 3. PPV4 – Clone_14 4. PPV4 – Clone_17 | 1. 209 2. 199 3. 119 4. 209 | 1. GQ387499 2. JX896319 3. GQ387500 4. GQ387499 | 1. PPV4 – Clone_17 2. PPV5 – IN273 3. PPV4 – Clone_14 4. PPV4 – Clone_17 | 1. 513 2. 392 3. 352 4. 513 |
| AB289986 | 1. EF172173 2. CU184692 3. AY285497 4. AB289986 | 1. *S. scrofa* – microsatellite sequence 2. Pig DNA sequence – Chr4 3. *S. scrofa* – microsatellite sequence 4. *Glypta fumiferanae* ichnovirus segment C9 | 1. 129 2. 123 3. 114 4. 0 |  |  |  |

^a^ Virus genome recovered by aligning the reads over the NCBI Viral genomes resource.

^b^ GenBank entries of the putative strains.

^c^ Virus – Short name. Full name and description are available in GenBank.

^c^ Covered bases of the viral genome derived from the whole alignments.

* The first three BLASTN results and the strain deposited in the NCBI Viral genomes resources (Ref_NCBI) are reported for comparison.
