# Supplementary material for: A viral metagenomic approach on a non-metagenomic experiment: Mining next generation sequencing datasets from pig DNA identified several porcine parvoviruses for a retrospective evaluation of viral infections
Source: PLoS One. 2017 Jun 29;12(6):e0179462. doi: 10.1371/journal.pone.0179462 (PMC5491021; doi:10.1371/journal.pone.0179462)
Supplement: S3 Table — (DOCX) [file pone.0179462.s003.docx]

**S3 Table. Viruses identified in the two libraries (LibP and LibN) by using the sequence assembly approach.**

| **Library** | **ID ^a^** | **GenBank** | **Name ^b^** | **Contig length** | **Contig coverage (%) ^c^** | **Virus genome ^d^** | **Virus coverage (%) ^e^** | **E-value ^f^** | **IM (%)^g^** |
| --- | --- | --- | --- | --- | --- | --- | --- | --- | --- |
| LibP |  |  |  |  |  |  |  |  |  |
|  | 585-286 | GQ906197 | *Babyrousa babyrussa* endogenous retrovirus class E | 287 | 98.3 | 1258 | 22.6 | 1E-131 | 97.19 |
|  | 784-271 | FJ600534 | PERV – WTR-23 | 272 | 91.2 | 1306 | 18.9 | 5E-120 | 98.79 |
|  | 3549-193 | DQ084474 | PERV gamma2 | 295 | 56.9 | 940 | 18.0 | 4E-71 | 96.45 |
|  | 4670-86 | KC701309 | PPV2 – 146PL | 423 | 100.0 | 3099 | 13.6 | 0 | 99.05 |
|  | CONTIG5 | FJ600533 | PERV – WTR-22 | 448 | 32.8 | 1153 | 12.9 | 9E-65 | 97.99 |
|  | CONTIG4 | JQ860245 | PPV2 – WB720I | 399 | 99.0 | 3099 | 12.7 | 0 | 100 |
|  | CONTIG2 | GU938301 | PPV2 – YH14 | 577 | 100.0 | 5619 | 10.3 | 0 | 99.83 |
|  | 5286-56 | FJ600523 | PERV – WTR-5 | 312 | 39.4 | 1221 | 10.0 | 8E-54 | 99.19 |
|  | 4749-82 | KC701292 | PPV2 – IIISRB | 311 | 99.4 | 3099 | 10.0 | 2E-148 | 97.76 |
|  | 5262-57 | KM259933 | PCV2 – ZJ-R | 327 | 19.6 | 694 | 9.2 | 1E-11 | 89.06 |
|  | CONTIG7 | GU938300 | PPV2 – JH13 | 486 | 95.9 | 5444 | 8.6 | 0 | 96.78 |
|  | 32-445 | GU938300 | PPV2 – JH13 | 446 | 100.0 | 5444 | 8.2 | 0 | 97.54 |
|  | 209-339 | GU938300 | PPV2 – JH13 | 340 | 99.1 | 5444 | 6.2 | 3E-162 | 97.64 |
|  | 310-318 | GU938300 | PPV2 – JH13 | 319 | 100.0 | 5444 | 5.9 | 5E-165 | 100 |
|  | 4433-99 | GU938300 | PPV2 – JH13 | 305 | 99.7 | 5444 | 5.6 | 2E-148 | 98.36 |
|  | 1-616 | HQ291308 | PBoV-H18 | 616 | 48.7 | 5267 | 5.7 | 2E-141 | 97.35 |
|  | CONTIG6 | HQ536015 | PERV-C | 343 | 99.4 | 8904 | 4.1 | 5E-116 | 89.04 |
|  | CONTIG3 | JX896319 | PPV5 – IN273 | 240 | 93.3 | 5788 | 3.9 | 1E-95 | 95.54 |
|  | 812-270 | JX896321 | PPV5 – IA46 | 271 | 82.7 | 5805 | 3.9 | 3E-107 | 98.67 |
|  | 2628-211 | AF033811 | Moloney Murine Leukemia Virus | 500 | 62.8 | 8332 | 3.8 | 5E-162 | 100 |
|  | 2892-208 | GU938300 | PPV2 – JH13 | 209 | 100 | 5444 | 3.8 | 5E-104 | 100 |
|  | CONTIG1 | KR709268 | PPV6 – KSU7-SD-2014 | 397 | 40.0 | 6148 | 1.8 | 1E-49 | 98.00/100^*^ |
|  | CONTIG7 | GU938300 | PPV2 – JH13 | 486 | 12.8 | 5444 | 1.1 | 6E-17 | 95.16 |
| LibN |  |  |  |  |  |  |  |  |  |
|  | CONTIG9 | FJ600523 | PERV – WTR-5 | 364 | 100.0 | 1221 | 29.6 | 2E-179 | 98.35 |
|  | CONTIG2 | KM259933 | PCV2 – ZJ-R | 334 | 49.4 | 694 | 24.1 | 3E-23 | 79.76 |
|  | TR870 | GQ906197 | *Babyrousa babyrussa* endogenous retrovirus class E | 201 | 99.5 | 1258 | 16.2 | 3E-85 | 95.59 |
|  | CONTIG7 | JX896319 | PPV5 – IN273 | 304 | 94.4 | 5788 | 5.0 | 3.0E-122 | 94.77 |
|  | TR3007 | KF999684 | PPV6 – SC | 242 | 98.8 | 6136 | 3.8 | 3.0E-101 | 95.40 |
|  | CONTIG6 | AF033811 | Moloney Murine Leukemia Virus | 501 | 62.7 | 8332 | 3.8 | 5.0E-162 | 100 |
|  | CONTIG8 | GQ387499 | PPV4 – Clone_17 | 221 | 90.9 | 5905 | 3.4 | 1.0E-99 | 100 |
|  | TR546 | HQ536009 | PERV-B | 260 | 80.4 | 9058 | 2.3 | 4.0E-91 | 96.21 |
|  | TR546 | HQ536009 | PERV-B | 260 | 79.6 | 9058 | 2.3 | 5.0E-90 | 96.17 |

^a^ Contig identifier.

^b^ Virus – Short name of the isolate. Full name and description are available in the corresponding GenBank entry.

^c^ Percentage of contig aligned nucleotides.

^d^ Virus genome size in nucleotides

^e^ Percentage of viral aligned nucleotides.

^f^ BLASTN Expected value of the alignments.

^g^ Percentage of identical match (IM) in the aligned regions.

^*^ BLASTN identified two matched regions on this entry suggesting a potential circularized genome or a chimeric contig: contig1 nucleotides 286-397 and 24-70 matched entry KR709268 nucleotides 1-112 (100% IM) and 6148-6103 (98.00% IM), respectively.
