# Supplementary material for: A viral metagenomic approach on a non-metagenomic experiment: Mining next generation sequencing datasets from pig DNA identified several porcine parvoviruses for a retrospective evaluation of viral infections
Source: PLoS One. 2017 Jun 29;12(6):e0179462. doi: 10.1371/journal.pone.0179462 (PMC5491021; doi:10.1371/journal.pone.0179462)
Supplement: S4 Table — (DOCX) [file pone.0179462.s004.docx]

**S4 Table.** List of pigs (identified by each line in the table) that were individually PCR amplified to detected the presence of different parvoviruses using primers reported in Table 1.

| **Library/DNA pool** | **Slaughtering batch^1^** | **Slaughtering year^2^** | **PPV2^3^** | **PPV4** | **PPV5** | **PPV6** | **PBoV1-H18_1-316nt_317-616nt^4^** | **PBoV1-H18_317-616nt** |
| --- | --- | --- | --- | --- | --- | --- | --- | --- |
| LibN | 1206 | 2003 | No | No | No | Yes | NA | NA |
| LibN | 2009 | 2001 | No | No | No | Yes | NA | NA |
| LibN | 0802 | 2001 | No | No | No | Yes | NA | NA |
| LibN | 0404 | 2002 | No | No | No | Yes | NA | NA |
| LibN | 2409 | 1997 | No | No | Yes | No | NA | NA |
| LibN | 0107 | 1998 | No | Yes | No | No | NA | NA |
| LibN | 2907 | 1998 | No | Yes | No | Yes | NA | NA |
| LibN | 2907 | 1998 | No | Yes | No | No | NA | NA |
| LibN | 0704 | 1999 | No | No | Yes | No | NA | NA |
| LibN | 0505 | 1999 | No | No | Yes | Yes | NA | NA |
| LibN | 2907 | 1999 | No | Yes | No | No | NA | NA |
| LibN | 1908 | 1998 | No | Yes | No | Yes | NA | NA |
| LibP | 3004 | 2003 | No | No | No | No | Yes | Yes |
| LibP | 1505 | 2003 | No | No | No | Yes | No | No |
| LibP | 1506 | 2000 | No | No | No | Yes | No | No |
| LibP | 2510 | 2007 | Yes | No | No | No | No | No |
| LibP | 1111 | 2004 | No | No | No | Yes | No | No |
| LibP | 2301 | 2003 | Yes | No | No | No | No | No |
| LibP | 1102 | 1998 | No | No | No | Yes | No | No |
| LibP | 1111 | 1998 | No | No | Yes | No | No | No |
| LibP | 1111 | 1998 | No | Yes | No | No | No | No |
| LibP | 1706 | 1998 | Yes | No | No | No | No | No |
| LibP | 0306 | 1998 | Yes | Yes | No | No | No | No |
| LibP | 1908 | 1998 | No | No | No | Yes | No | No |
| LibP | 1908 | 1998 | No | No | Yes | Yes | No | No |
| LibP | 1612 | 1998 | No | Yes | No | No | No | No |
| LibP | 1407 | 1999 | No | Yes | No | No | No | No |
| LibP | 0903 | 2000 | Yes | Yes | No | No | No | No |
| LibP | 0903 | 2000 | No | Yes | No | No | No | No |
| LibP | 2004 | 2000 | No | Yes | Yes | No | No | No |
| LibP | 2004 | 2000 | No | No | Yes | No | No | No |

^1^ Code of the slaughtering batch that identifies contemporary animals grown in the performance testing station.

^2^ Slaughtering year of the corresponding pig

^3^ “No” indicates that PCR did not produce any amplified fragment (negative); “Yes” indicates that PCR produced an amplified fragment of the expected size (positive).

^4^ NA: Not amplified.
