# Supplementary figures and images for: A viral metagenomic approach on a non-metagenomic experiment: Mining next generation sequencing datasets from pig DNA identified several porcine parvoviruses for a retrospective evaluation of viral infections
Source: PLoS One. 2017 Jun 29;12(6):e0179462. doi: 10.1371/journal.pone.0179462 (PMC5491021; doi:10.1371/journal.pone.0179462)

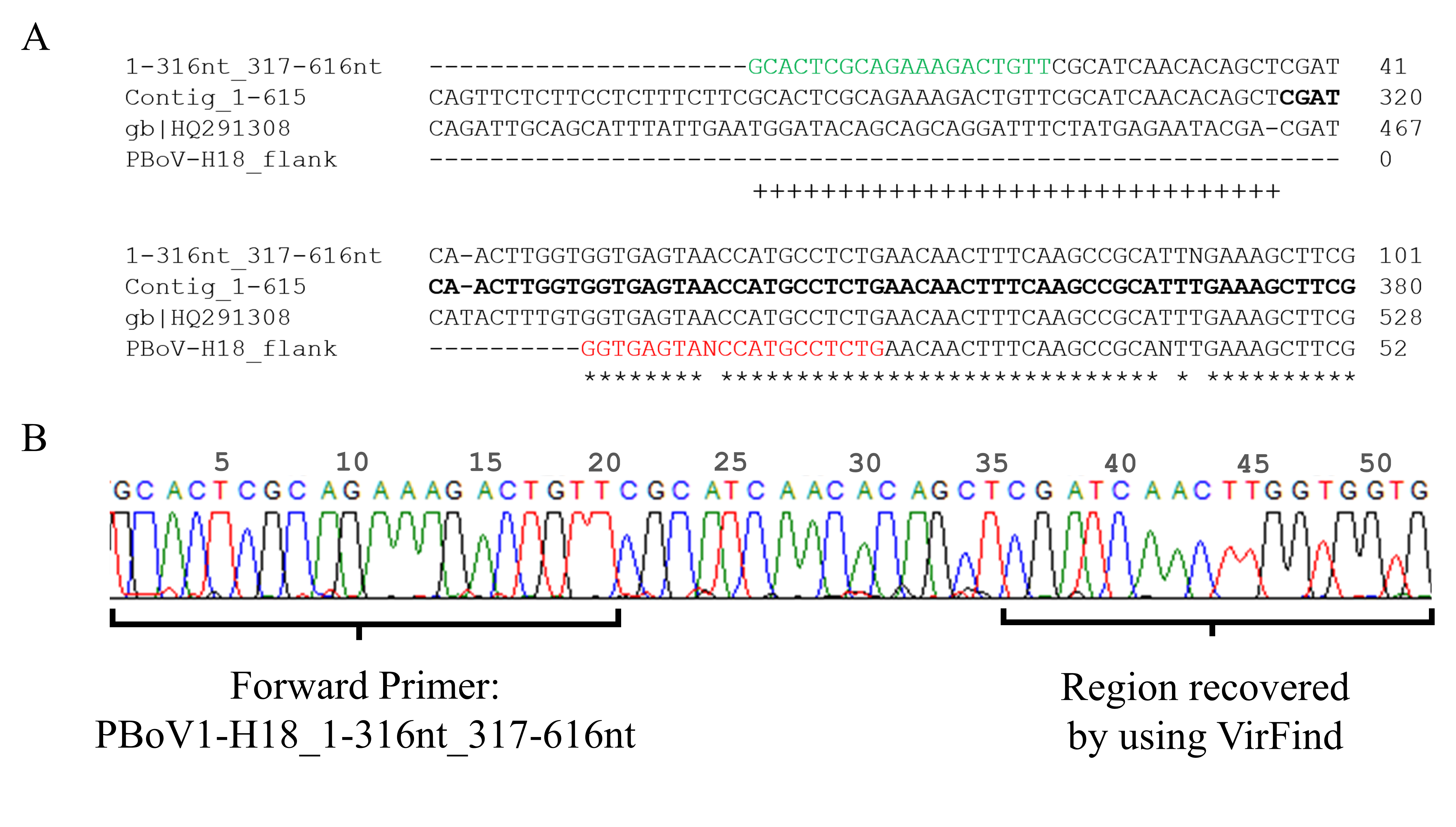

Supplement: S1 Fig — (A) The forward primers of the PBoV1-H18_1-316nt_317-616nt and PBoV1-H18_317-616nt primer pairs are highlighted in green and red, respectively. Nucleotides of the Contig_1–616 aligned by using VirFind are in bold. “+” indicates a sequence identity between the portion of the Contig_1–616 not aligned by using VirFind and the DNA region sequenced by using primer pair PBoV1-H18_1-316nt_317-616nt (100% identity). Sequence identity between these 35nt and the PBoV1-H18 genome (GenBank: HQ291308) is equal to 20% indicating that the portion upstream the region recovered by VirFind could be (at least for these 35 bases), very dissimilar from the reference HQ291308. “*” indicates a sequence identity among the Contig_1–616, the reference and the two obtained Sanger sequences. (B) Sanger sequence of part of the Contig_1–616. The primer and the region aligned with VirFind are highlighted. (TIF) [file pone.0179462.s005.tif]
