# Supplementary material for: A viral metagenomic approach on a non-metagenomic experiment: Mining next generation sequencing datasets from pig DNA identified several porcine parvoviruses for a retrospective evaluation of viral infections
Source: PLoS One. 2017 Jun 29;12(6):e0179462. doi: 10.1371/journal.pone.0179462 (PMC5491021; doi:10.1371/journal.pone.0179462)
Supplement: S1 File — (DOC) [file pone.0179462.s006.doc]

**S1 File. List of contigs generated for the LibP and LibN datasets with the sequence assembly approach.**

>TR277_LibN

CTTAGAGCCGAGCGTGATTCGCCCAGGAGAGCTGGTCGACCCCGTGGGCCCTCAGAGCAA

GGCCGGAGTCACCCAGGACAGCTGGTCGACCCCGCAGGCCCTCAGGCCCGGGCCCGAATC

GCCCGTGAAATCTGGTCAACCCCGCTGGCACGCGGACCCGGGCACGATTCGCCCGAGAGA

GCTGGCCGATCCCGCGGCCCTTGGACCCGGGCCCGAGTGCCCTAGACAGCAGGTGGACAC

CGAGGGCCGTCGGATCCAGTCCTTGGTCACCCGGGACAGCTGGTGGAACCCGTTGGACTT

CGGACCTGG

>TR277_LibN

CTGGCTCCGAGTTCCCAGGGTGTCGACCATCTGTCCCTCGATTTGGGCCCGGTTCCCAGG

TCCCATGGGGTCAACCAGGTGTCCCGGGCAACTCGCATTCAAGTCCGAGGGCCCGCGGGG

TCGACCAGCTCTCCCGTTCCTCTCGGGCCCGGGTCTACTGGTCCGGGGGATCAACGAGTT

TTCCCAGGCCACTCGGGCGTGTGTCAGAGGTCCCGCGGCTTCGACCAGCTCTCCCCGGGC

GAATCACGCTCGGCTCTAGGGCCAACGGGGTTGACCCGTGGTCCTGGGCGACAAGGGCCA

GAGCCCTATGGCCTGCG

>TR546_LibN

AGCAACTGTAGCTCTGATATCAGCTCCTGAAAGGCCAGTAAAAGAACAATCCCCCTCGTC

CGGGCTCGGAAGAGGCGACACCCCAAATCACTCACGAGAAGCGGTCTTGATGCAAACAGC

AAGAGGATTTTTATTCCAAGCGCGCTGGGGCCCACAGTCGTAGGCCACGCAGGGTAGAGG

ACTCGGCGGCGTGTGCGGAGTCGGGACAGCTTTTATGGGGTTCACAACAAAGCCCACGCT

TCGTGGACCAATTAATTTAA

>TR870_LibN

GTATATAGGAACTCCCAGTTGCTCCCCTAGTGACAGCGGGAGCAGGAAGAAGGAAGGTCT

GATTGTTCCCAGTACACATGCCCCAGCCAATTCGGGGGAGTATGGAATAGCCATTTTTCA

CAAATCCAATATAATCCCTTTGGTGCCCGCCTTTGGATATTAGTACTGATGTCTTCCCAT

GCTTGTCGAAGGAGGGAAAAG

>Contig2_LibN

CCTCGCTCCTGGGCCCGAGTTACCCGGGACAGCGGGTCGACCCCGCGGGCTCGGAGCCAG

GCCGGCGTCGCCCAGGACAGCTGGTCGACCCCGCGGTTCTTCAGACCGGGGGCCGAGTCG

CGGGGGACAGCTGGTCGACCCGTGGGCCTCGGACACGGGCCCCAGTCGCCCGGGACAGCT

GGTGGATCCCACTAGCCCTAGGACACAAGCGCGAGTGGCCCAGGACAGCTGGTCCACCAC

AAGGGCCCTCGGATCCTGGACCGAGTCACCCGGGACAGCTGGTCGACCCGAGGCTTTCGG

ACCCGTCGCCCAGTCACCCGGGACAGCTGGTCGA

>Contig7_LibN

GCCGGCGGCGTACGCCGCCGGCCTGCTAATAAAAACAATAGCGGTGGGGCTTCGCCATCT

TTGACAGATATGTTGCAATCAACTTCCGGGGGTGGGACTTCCGGGGTCAAAGGTCACTTC

CGGGTCAAAGGTTTTTCTTCCGGTGACGTCACGGGATGTGACGCAGTACAGACCGACGAG

ATATTTGGCGCTTTTCATACGTCATTTCCGGGGGAGGAACTCATTAGCATAATTTATGCA

AAGTGGGAGGAGCTTATTGGCTCCTCCTACTTTGCATAAATTATATTTATGAGATGTAAA

TTAA

>Contig8_LibN

CAGGCCTCTCGCCGGTCGCCCCTGACGTCACATCCGCTTCCGGGTCAAAGGGCGGGGTCA

AAGGTCAAAGGTCTTCATACGTCATATCCGCTTCCGGGTCATGACCTTTGAACCGGAAGT

ACCGGAAGTGACCCGGAAGTACCGGATGACCTCTTGCGCAATCTCTGAGAAAGAGAGAAT

CTTTTTCGAGTGTTGGGCGACCGGCTTCGCCGGTCGCCTAA

>Contig9_LibN

CAGCGCCAGCGCCAGCGCCAGCGCCAGCGCCAGCGCCAGCGTAGGGAAGTGTGACCGAAA

CCAGGTGAGCCGAGTTAGGCAGGGCTGGTTGGTCTCTTCAGGAAATCTCGGAGGGTGTTC

CCTCGTGGGGGCCTGGCTCAAGTGTCTCTCGACCTCACTGCTGCGACTGTGGTAATACGT

GCATTTGGAGATGCCACCCTGGTCAGACAGCATCGCAGTACCGCAGGTGACACTGAAATA

TACTTCTCAATAGAAGAAGAAGTCATTGAAAAGAACGAGCTCAAGGGCCTAAAGGAGTCA

CTGTACGCATTCATGCCTTCCTATAGATTAACGAACGGAATTGCTAGATAAGGCAATGAT

GAGG

>Contig6_LibN

CCTATCAAGAAATCAAGCAAGCTCTTCTAACTGCCCCAGCCCTGGGGTTGCCAGATTTGA

CTAAGCCCTTTGAACTCTTTGTCGACGAGAAGCAGGGCTACGCCAAAGGTGTCCTAACGC

AAAAACTGGGACCTTGGCGTCGGCCGGTGGCCTACCTGTCCAAAAAGCTAGACCCAGTAG

CAGCTGGGTGGCCCCCTTGCCTACGGATGGTAGCAGCCATTGCCGTACTGACAAAGGATG

CAGGCAAGCTAACCATGGGACAGCCACTAGTCATTCTGGCCCCCCATGCAGTAGAGGCAC

TAGTCAAACAACCCTTAAGCAGAATTTCTGTCATCATGGACAGCACGGAACGGGTGAAGC

TGCGCCAGTTCTGCTCACTGCCGGTCAGCATCGCCGCCATATTCTGTGCAATACCATCAA

AGGTCTGCGTGGCTGCACTTTTTACCTGCGACATACTGTCCGTGGCGCTCTCTTCCCACT

CACTCCAGCCGGACTTCAGGG

>TR2520_LibN

TTTGTGCACGAGATGCGAGCATGGGCCGCGAAGCGGCCCATGCACGCAAATCTCGAGTGC

ACAAAATAAAAGTGTGATGTTTTCAAACTCTAAGACAGACATTATGCAATCCAATATGTC

TACTCTTCCGGGTCAAAGGTTATACTTCCGGTTCAAAGGTTACCGGATACACGTCATCCA

AAATGGCTGTCGTTCCGGGTTGTAGGTCATAACGGTTTTGGCGCGCGGAAGTGACGTTTT

TTGAATTTAGGCGCGCGGAAATGACGCCGTTTGTCGCTTACGTGTCAGCGACCGGCGAGA

AAGATGG

>TR3007_LibN

AAAATACGTCAATTTCTGATGTAATTCGTGTTTGAGAAACGCATATTTTCTTGCCGGTCT

GAGTGACACCACCTATGACATCATATAAATTTGATTACGTAACTTCCTCTTTTTACTTCC

TCTTTTTTGATTACGCAATATCACAATTCTAGCAGTCGACTATTACACAATATCACACTT

TGAGACCCTGACCATCACACTTTTATTTTGTGCACTCGAGATTTGCGTGCATGGGCCGCT

TA

>Contig1_LibP

TCGAGATTTGCGTGCATGGGCCGCTTCGCGGCCCATGCTCGCATCTCGTGCACAAAATAA

AAGTGTGATGTTTTCAAACTCTAAGACAGACATTGTGCAATCCAATATGTCTACTCTTCC

GGGTGAAAGGTTACACTTCCGGTGCAAAGGTTACCGGATACACGTCATCCAAAATGGCTG

TCGTTCCGGGTTATAGGTCATAACGGTTTTGGCGCGCGGAAGTGACGTTTTTTGAATTTA

GGCGCGCGGAAATGACGCCGTTTGTCGCTTACGTGTCAGCGACCGGCGAGAAAGATGGCG

TTTTTCATTTACGTTTTGATGACGTTGCTGTTCTCTGATTGGTTAGTTTTTCAAGTCCTC

ATTTGCATATTTAATGGACCAATAGGATTTACTTCTG

>Contig2_LibP

CAACAGCCTCAGCACTCTCAGCAGGCCTCTCTTCAGGTGCCGGCCGAGATCGCTCTCCGG

CTGCAGACCCAGGATTGTCGATACGAGCGACCTGAATCTCTTTCGGACGGTCTTCCTCTT

CTGCCTTGTAGGAGGCATCTAAGGAATTACGGATGTCTAGGTCAAGCTCTGCCCAGGTAC

CCCAGGGACTTCCCTTCGGGGGCTGAGGTCTATACCCGCCCGTAATCCACACCCTCATCA

CATCTCTCTGAGGTAAACGGACCTTGTCAGCGAACCGCTGTAGTCCAAAGAAGGCCCTCG

TGGCATCATTGTCCCCGTAAAGAAAATCTATCATGCTCCGCCTGACGCTATCTATCTCTG

GGTCCTCCCTATAGTCATCTACGACTGCCATAAAGAGCCTTCCCAGATCGAGCACTGTTC

GCGCTAGCTCGTCTCCCTTGGAGGTCTGGTGTCCCGCCATCCACCATTGCTTCGCCACCT

CCTTAATTTTATCCCAGAAACCACCGTGTTTGTTTATTGAGATAAAAAACTTGTAAAGCT

GTAAGAGAGCCTGGCGCATGCGCGGGTCATAGCCAGG

>Contig3_LibP

TTGCATAAACTATGCTAATTAACTGCATCACAGGAAATGACGTATGAAAAGCGCCAAATA

TCTCGTCGGTCTGTACTGCGTCACATCCCGTGACGTCACCGGAAAAACAACCTTTGACCC

GGAAGTGACCTTTGACCCCGGAAGTCCCACCCCCGGAAGTTGATTGCAACATATCTGTCA

AAGATGGCGAAGCCCCACCGCTATTGTTTTTATTAGCAGGCCGGCGGCGTACGCCGCGGC

>Contig4_LibP

TGGGGGAATGAACGGCAGGGAAACTTTCTCCGTTTTAGTGGCTCCAGGTCCTGTACTGCT

GCCAGGTAATTTCGCTCCCCAGGTGGGCACCATAAGGGCATCTCCAGGTCTTTCTCCAAG

ACCTTCTCTCTCTGCTGTCACCAGAGGTCGAGGTTTTTTGACGTAACCACCAGGATTACG

GTAGTCTTTGTCCTCGTCAAATCTTTCATAAAAAGAATCTTCAGCAGAGGACCGAGCAAA

GGCCATATCACCCAATTGGAAATGTACATTTCTCTCTACCTCCCCCTGGCGATTATAGTT

TTGAGTGGAGATGGTTGTAGCCTGTCTGGTAAACGGCATGAGAGAGTGCCGATTATTACT

AGTGCGATCACCGTAGGCGATCGCAGACATCTCATCAAA

>Contig5_LibP

CCTTGTGCAGTGGGTTAAGCAATCCAGCATTGCCTTGAGCTGTGGTGTGGGCTACGGATG

TGGCTTGGATCTGGCATTGCTGTGGCTGTAGCGTAGAACAGCAATGCCAGCTCTGATTCG

ACCCGAGTCGGGAACTTCCCTATGCTGCAGGTGGGGCCCTCCAATGCCAATCAGAGACTC

CATCTATCCATGCATCCCTCCATCCAACAAGGAAACAGTGGATTAATGGATGGCACATTC

ACCGCATCACAGAAATGGCCGCCATGCCCAGGGTGGGTGAAGGAGCTTCCCAATTTCAGG

GGCTGGATTGGTGCAGCTTGTGTGAGACAGAGACAGTGGCTGAACACTCTTTGTCTATGA

CAGACACCAAGGGGAAGCTGACCCTCTGATACCAGGGGCCCACATCCTAAGTCAAATGGG

TTTCTTCAGCCAATGCTTTGACTCCCCC

>Contig6_LibP

GAGCCATTGAGGCTTTCTGAGCCTCCGAGGTAGGATCAAAAGGGGTGAACCGCCTGAAGG

CTTCCATGAGCCTCTCAAGAAAAATCGAGGGAAGTTCATTCGGTCCCTGCATCACCTCTT

TTACCTTAGCCAAATTAGTGAGACCCGCCACCAGAGCCTGGTGATAGATTTTCAAGCTCT

CCCTACCTTCAGCCGTGTTGTAGTCCCAATGGGGCGAGTCAGGAAATCCCATGTCAATCT

CATTTTGCAACTGTGTGGGTCGCCCGTCGGCCCCAGGAACATTTTTTTTTTTAAGCCTCT

AACAGAATTCTCTCTCGCTCCCCGGTTGTGGAGAGTGTCTGTA

>Contig7_LibP

ACCTTTATTTGAACCCTGGCTTTCTAGTCGGACCGGAAGTCGCCGAATGATGACGTAGTT

TCACTTCCGCCACACTCAAGATGGCGGCGGTTCCGGTTCCGGTTTCCGGTTTCCGGTGAG

TCAGCACTTCCGGGTTTGGCCACGCCTCCGGATGTCGTCATACCGAGCCGACCGGACCGA

CAGGATAAGTGTCGAGAATAAGGAAGCGCGAGTCATTCTTCGGGTGGACACTGAAGAGGG

CGGAACTCGTCATGGCAGGTCTCCTGTACCTCGGCGTGCTCAAGCTGTACCGGGGCGACT

TGGAGCCCGTGAGATGGCTGCAAGGAGACCGTATGGCGAACAGGAACATGCCGGCACCCG

AGGACTGGCCGACTGAACAGCTCTGTGACAAGTGGGGCCGAGAGGTATTCAGGTTCCTGG

TCAGCGTCTTCGAGCTGATTGACGAGACGCTGCAGGTAACCTACGACAACTACTTCAGGG

AGAGAA

>1-616_LibP

CGGGCGCGCTGCGCGCGCGCGCGAAGGCGGGGCGATATAAGCAATGCGCATGCGCATAAG

CATCAAACATGGTGTCCTAGTGGTGAAATAAAGTTATAACAATACAAAATATTATATAAT

TACGTCATATCCGGTCATGCGCAAAATCATATACGGAACTAAAAGTGCACAATCATCGGA

AGAACCTTGAAAGGTCGTTATGTAATTTCTAATAACTAACTAATGGGTGGAGTTATGAAC

TGTATAAAAGGAGAGAACACCAGTTCTCTTCCTCTTTCTTCGCACTCGCAGAAAGACTGT

TCGCATCAACACAGCTCGATCAACTTGGTGGTGAGTAACCATGCCTCTGAACAACTTTCA

AGCCGCATTTGAAAGCTTCGGGGGAATTGCCTATACTTATATTCTGAGACTTCCTAAATT

TCCTACGAATAATTATCATAATATGCTGCAGCGATGGTTTGGGGATAGCGAGCATATCTA

TTGCATGAAAGAGAGGAATGCGGAAAGCACTATCCAGACTTTCCAAATGATTGCTCCGAC

TTAGCTGATTACCAGAAAAAATCATACGAACACAACATTGCTATATTTGCTGAAACCGCC

GTAAAAAAGGTGTTTG

>32-445_LibP

GTCGAGAATAAGGAAGCGTGAGTCATTCTTCGGGTGCAGAGTAAGAAAGCTTTATTGAAA

CCTCTTTATACACGATGCGCGCGTCCCTCTGGGCGAAACGCTTCTACAGGTAAAGAATAT

CGAGGGTAATCATTGGAAACATGCGAACGAGTAAGGAGAAACGGGGGGTCGCCGGACTCC

GTGTAAGGCATGGGAGGCGGAGGTTCCAAATTATGCCGTCTAGTGTGCTTCCGCGGTTCG

CACACCCACTCCATAGTGTAGCTCACAGTGAACATGGCATACTGATTCAAAGCAGGCGGC

AGAAAGGTTCGTTGGTCCAAGTCCGCCGGTGGCGGACCCACCTGCGGCTGCATACGAAAA

ACACTTGGGGCGGAGGATCCACCAGCGCCCAGGCACCGAGATTATTTTTCTCAGACATGA

AAGAGCAATCAGTGTATGGCTTCTTA

>209-339_LibP

GCATTGATCCCTCTCCGCCCGCCAGAGTTGTTTCTTCTATTGCCCCAAATGGATCTGTCC

ATGGCATCCATGAGGTTCTCCAGCGGGGGCCGGTCACCCGGCTTCCACGCGTCGGCAGCG

CTCATGGCCGCTTACCTGGAAGGAGATCGGAGTCCGAGGTTAGAGCCACGCTTGCCAAGG

TCGCAAGACCGTGTTCGACGGTCGGACGCTTCCGCCTATAAGGGAAAGGCAGGGATATGT

AGTACCAGTTGTCAACGTTGTGCCGAGCTCTGATGCACGCCGACTCGCTCCGAGACACCG

AGGACCGTCTGGCGTCCCAGGACCTGAGGATTCTCTGCAG

>310-318_LibP

GTGCACATGTCACCGTTAGAGGTGATCACCACGGGAGCAGGTATAAACTGCTCTGAAGGT

TTACCCTTAATGTCAATCCTTATCTCGCTGCCCCCCAGTATGGCCTTAGCGGCCTCTACA

AACTTCTCAGACATCTTCCCTTCCTCCCACCAAATCAGGGATTGAGAGGCACAGTCACTG

AACGGGAAATTCTCATTGTTCCAATTCACGCAGCCGAAGCAGGGAGCTAGTTTGGCGAAT

GCGCCGGCTAACAGCGTCTTGCCAGTGTTAGCGGGTCCGAACAGCCAGAGGGCTCTCCTC

TTTTGATGTCCCCCCTTGG

>585-286_LibP

TATTCTTTCTGGGGGCCATTCATCATCTTTCCAGTCTCCAATTTGGAGAGCCCTCCGGTC

TCTCTGGGCACCCTGTGCCTATATATAGGAACTCCCAGTTGCTCCCCTAGTGACAGTGGG

AGCAGGAAGAAGGAAGGTCTGATTGTTCCCAGTACACATGTCCAGCCCAATTTGGGGGAG

TATGGAATAGGCCATTTTTTTACAAATCCAATATAATCCCTTTGGTGCCCGCCACTGGAT

ATTAGTACTGATATCTTCCCATGCTTGTCGAAGGTGGGAAAAGTTAG

>784-271_LibP

CTGAGGTGCAAAGGAAGACAAGTTTCAGTCCCACTTCTGGATCCTCAGTTCTGATTTGTG

AAGGCCAACAGGTGCTGCCCTGAAATTCATTATCACAGCCTTTTATATATGGAGTGAAGG

AGTTACAGAAATCCCTCAACCTTTCAAGGTCATTTAAACCAAATTAGTAGCAGAATTAGA

CCATATCTTTCCAGGGACATTGTAGATTTCCCTTGTGGTGAGATGATGACTCTAAGCAAA

TTCCATCTATTGCTACCCCACCCACCCCAGGG

>812-270_LibP

GTAAAATTCTAGTGTTGGGCGACCGGCTTCGCCGGTCGCCTAAGCCTGGAGGAATTTTAC

ATTCTCTATCTCATAGATTGCGCAAGAGGTCATCCGGTACTTCCGGGTCAACTTCCGGTA

CTTCCGGTTCAAAGTCATGAACCGGAAGCGGATATGACGTATGAAGACCTTTGACCTTTG

ACCCCGCCCTTTGACCCGGAAGCGGATATGACGTCAGGGGCGACCGGCGAGAGGCCTGGC

GCTTTTCAAATTCAAAAAAAAGAGGAAGAAC

>1188-251_LibP

ATACCTACCATCTAGATTATACCATTAACATTTTACTGTGCTTGCTTTATCACGTATCTA

TCCATCACTCTCTACCTCAGATGTGTAAAGATTTTAATTCTGACTATGCAAGGAAAGATT

AAATTGATTTTGTTGGGATCAGTTTGAAGCCTAGTCAAGTTATATCTTTGCCGTCTTTTT

TTAAAAAATTGTTTAAATTTTTTAATTTTTCGCCTTTTTTTTTTTAGGGCCTATACCCGC

ACAGCGGGATTG

>2628-211_LibP

CCTGAAGTCCGGCTGGAGTGAGTGGGAAGAGAGCGCCACGGACAGTATGTCGCAGGTAAA

AAGTGCAGCCACGCAGACCTTTGATGGTATTGCACAGAATATGGCGGCGATGCTGACCGG

CAGTGAGCAGAACTGGCGCAGCTTCACCCGTTCCGTGCTGTCCATGATGACAGAAATTCT

GCTTAAGGGTTGTTTGACTAGTGCCTCTACTGCATGGGGGGCCAGAATGACTAGTGGCTG

TCCCATGGTTAGCTTGCCTGCATCCTTTGTCAGTACGGCAATGGCTGCTACCATCCGTAG

GCAAGGGGGCCACCCAGCTGCTACTGGGTCTAGCTTTTTGGACAGGTAGGCCACCGGCCG

ACGCCAAGGTCCCAGTTTTTGCGTTAGGACACCTTTGGCGTAGCCCTGCTTCTCGTCGAC

AAAGAGTTCAAAGGGCTTAGTCAAATCTGGCAACCCCAGGGCTGGGGCAGTTAGAAGAGC

TTGCTTGATTTCTTGATAGG

>2892-208_LibP

GATCGAGATGGATAAGATGGGATATCGTTCTTTCATGTCCACCTCTCAAGGGGTGCTGCA

AGTGAAGAACGCTCTGAACCTTGCACGCAGGGAACTCGTAGCAGGGAACAAACTGTTGGA

GAGCATAGTGAAAGGTGCTGACCCCTGGGTACCTGGAGTTGTGAATGCCGTAGCCAGGCT

CTTCCACATTAACGGCTATGACCCAGAAC

>3549-193_LibP

GTCGTCGTTTCTCTTCTCTTTTCACAGCAAGAGCCATGATTAAAGGAGACGGGGTTCTGT

CAATTTGAGTTGGGCCGATCCATCAGTAGAAAAGGAAATTTGTGCCCCCATTTTAGCCAG

CAGGTCTTTTCCATCAAGGGGACTGAGCAGTCAAGCAGTTAAAGAAATTCGTGGACTTGG

AGAATAGACTTGTGGTTGCCAAGGGGGAGGGGCAGGGAGTGGGATGGATGGCGTGCTTGG

GTTAACAGATGCAGACTATTGCCTTTTTTTTTTTCTTCTTTTTGCTATTTCTTTG

>4433-99_LibP

CCGGCTGGTGCACTCGGGCAGGAACAGGTAACCCAAAGCCTCCCGGGCAGGAGCTCCGTA

CCTTGGTTCCTCCTCGACGTCCGTCTCAGAGTCTGAGTACTCGGTGAGGCGACGTTTCTT

TTGACGCTCTCGGGGCATGCGCGTTGGAGTATTCTGCGGGGGGTCGGGGAACCACTCCTC

CTCCGAGTCCCAGGTGTCCCTCTCCTCCCGTATGGGCGGAAGGGCGGAGCTTTCTATCTT

TGGCGCTGTGGTCGAAACTTGGCAGGGTGGAGCTACCGCGGCTACGGCCAGTTGATCTAT

TTCGG

>4670-86_LibP

GTACATCAGACAAACGAGCGAGACAAACGTGCATGACTCCGGAACAGGTGGCATCCTGAT

ATTCGAAGACTCCGAGTACACCTTTCCGTATGTCATTGGGCATGCTCAAGAGGGAAACCC

TGGTGCACTGAGTATTCAGTGGTACAACCCTCCCCAATACGCCTATTTTACGGGTTTTAA

TCCTATTGCTTGGGACCATGCAAACGGTACCATTAAGTATCAGGTACATCCTAGCGCGGA

CACGGAGTTCTTTGTTCTGGAGGAACACGCTGCCCAAATACTGCGCAGCGGTGATGGTAC

CTCCTTTGCTTACGAGTTTCCCAGTCTCGAACCTAAGCGACTGGGCAGCCGTATGGGCAC

GTTGAACCTCCGTCATAACCCTGTTCTGCCCAGCAGACTGGCGATTTATTTGGGACAGGA

TGG

>4749-82_LibP

TACCCTATATTCACGGGAGGGGGGCGGACAGTCTGATGGGGAAAGAGCTTGCAGATGCCG

AGGAAGCGGGGAAGATACTCGATAGTTATGATCAGTTAGTGGCGAATGCCGCTAGGGGGC

TGTGGAGAGCTAAGGACACGTTAGCGGATCTCATTGGTGGGGAGCTGGACAAAGTCTTAC

CGCCCGATCCTCCCGTACAGAGCGAGGGGGAGGAGTCCCAGAAGAGACCTCGTGAGGAGG

AGGATCCTCCGGAGAGCGCCGACTCCGCCCCCAAAGCCCCGCCCGCCAAACCAGAAACCT

AGACGTCCGAG

>5262-57_LibP

CTAGGACACAAGCGCGAGTGGCCCAGGACAGCTGGTCCACCACAAGGGCCCTCGGATCCT

GGCCCGAGTCACACGGGACAGCTGGTCGACCCGACGGGCCCTTCGGCATGGGCCCGAGTC

ACCCCGGAGAGCTATTTAACCCTGCGGACAATCGGACCCGAGCGCGACACGCCCGGGAGA

GCTGAGAGTCCCTGCGGGCCCGCGGACACAAACCCGAGTGGCCCGTGAGTGCTGGTCGAA

CCCGCGGTGCCTTGGACGCAAGCGCGAGGGGCCCGGGACAGCTGGTCCACCCTTCAGGCC

CTTGGAGCTTCGCCAGAGTCGGCCGGG

>5286-56_LibP

AGACACTTGAGCCAGGCCCCCACGAGGGAACACCCTCCGAGATTTCCTGAAGAGACCAAC

CAGCCCTGCCTAACTCGGCTCACCTGGTTTCGGTCACACTTCCCTACGCTGGCGCTGGCG

CTGTACTGCAAAGGAGAGAGCCTCATCGCTCAAGGCAAAGAGGAACTATGCTAGGGCGAT

TGAGGGAAAAGGAAAGCACAGGGGATTGCGCCGCTGCCAGCCTTCATGCCACCCGCTGCC

CCACAGTCCCGCCTCGGCACCAGGCTGACTCTCTCACGTACCTAAAACGGTGGGAGGCGG

GGCTCCGCAAAT
