# Supplementary material for: A viral metagenomic approach on a non-metagenomic experiment: Mining next generation sequencing datasets from pig DNA identified several porcine parvoviruses for a retrospective evaluation of viral infections
Source: PLoS One. 2017 Jun 29;12(6):e0179462. doi: 10.1371/journal.pone.0179462 (PMC5491021; doi:10.1371/journal.pone.0179462)
Supplement: S2 File — (DOC) [file pone.0179462.s007.doc]

**S2 File**. Alignment of the PCV2 contig with BLASTN matched porcine genomic sequences.

| Dataset | query ID (contig) | subject IDs^1^ | % identity^2^ | | q.length^3^ | mismatches^4^ | gap opens^5^ | q. start^6^ | q. end^7^ | s. start^8^ | s. end^9^ | E-value^10^ |
| --- | --- | --- | --- | --- | --- | --- | --- | --- | --- | --- | --- | --- |
| LibP | 5262-57 | gi|288707504|emb|HE430474.1| | 96.33 | 327 | | 12 | 0 | 1 | 327 | 455 | 129 | 5.70E-150 |
| LibP | 5262-57 | gi|268779272|emb|HE255902.1| | 96.024 | 327 | | 13 | 0 | 1 | 327 | 202 | 528 | 2.65E-148 |
| LibP | 5262-57 | gi|268754310|emb|HE301064.1| | 96.024 | 327 | | 13 | 0 | 1 | 327 | 544 | 218 | 2.65E-148 |
| LibP | 5262-57 | gi|268966898|emb|HE259573.1| | 96.486 | 313 | | 11 | 0 | 1 | 313 | 231 | 543 | 7.43E-144 |
| LibP | 5262-57 | gi|269036928|emb|HE092449.1| | 95.37 | 324 | | 14 | 1 | 1 | 324 | 364 | 42 | 9.61E-143 |
| LibP | 5262-57 | gi|268902279|emb|HE002591.1| | 95.37 | 324 | | 14 | 1 | 1 | 324 | 224 | 546 | 9.61E-143 |
| LibP | 5262-57 | gi|269006791|emb|HE177596.1| | 95.062 | 324 | | 15 | 1 | 1 | 324 | 408 | 86 | 4.47E-141 |
| LibP | 5262-57 | gi|288635963|emb|HE479338.1| | 97.924 | 289 | | 6 | 0 | 1 | 289 | 380 | 668 | 2.08E-139 |
| LibP | 5262-57 | gi|268950875|emb|HE279831.1| | 97.611 | 293 | | 6 | 1 | 1 | 292 | 300 | 592 | 7.48E-139 |
| LibP | 5262-57 | gi|268891780|emb|HE047306.1| | 94.444 | 324 | | 17 | 1 | 1 | 324 | 357 | 35 | 9.68E-138 |
| LibP | 5262-57 | gi|268754306|emb|HE301060.1| | 94.136 | 324 | | 18 | 1 | 1 | 324 | 594 | 272 | 1.25E-136 |
| LibP | 5262-57 | gi|79965566|emb|CT364390.1| | 95.752 | 306 | | 13 | 0 | 22 | 327 | 748 | 443 | 1.25E-136 |
| LibP | 5262-57 | gi|269056048|emb|HE029483.1| | 94.136 | 324 | | 18 | 1 | 1 | 324 | 652 | 330 | 4.50E-136 |
| LibP | 5262-57 | gi|269041112|emb|HE085726.1| | 94.136 | 324 | | 18 | 1 | 1 | 324 | 559 | 237 | 4.50E-136 |
| LibP | 5262-57 | gi|268765006|emb|HE272786.1| | 94.136 | 324 | | 18 | 1 | 1 | 324 | 457 | 135 | 4.50E-136 |
| LibP | 5262-57 | gi|288712295|emb|HE436677.1| | 94.357 | 319 | | 17 | 1 | 6 | 324 | 619 | 302 | 5.82E-135 |
| LibP | 5262-57 | gi|268772881|emb|HE247166.1| | 95.13 | 308 | | 13 | 2 | 1 | 307 | 328 | 634 | 7.53E-134 |
| LibP | 5262-57 | gi|269038613|emb|HE099864.1| | 96.246 | 293 | | 10 | 1 | 1 | 292 | 300 | 8 | 3.50E-132 |
| LibP | 5262-57 | gi|288614170|emb|HE548276.1| | 98.155 | 271 | | 5 | 0 | 1 | 271 | 271 | 1 | 1.63E-130 |
| LibP | 5262-57 | gi|79828921|emb|CT274736.1| | 94.755 | 286 | | 12 | 3 | 1 | 284 | 285 | 1 | 4.60E-121 |
| LibP | 5262-57 | gi|79850152|emb|CT304582.1| | 95.817 | 263 | | 10 | 1 | 62 | 324 | 851 | 590 | 1.67E-115 |
| LibP | 5262-57 | gi|79839159|emb|CT196639.1| | 92.466 | 292 | | 21 | 1 | 33 | 324 | 1 | 291 | 2.79E-113 |
| LibP | 5262-57 | gi|79660894|emb|CT470548.1| | 92.632 | 285 | | 20 | 1 | 40 | 324 | 1 | 284 | 4.66E-111 |
| LibP | 5262-57 | gi|78644928|emb|CT040860.1| | 93.561 | 264 | | 16 | 1 | 61 | 324 | 851 | 589 | 4.70E-106 |
| LibP | 5262-57 | gi|268741933|emb|HE033777.1| | 93.822 | 259 | | 15 | 1 | 66 | 324 | 1 | 258 | 6.08E-105 |
| LibP | 5262-57 | gi|79694411|emb|CT268714.1| | 93.462 | 260 | | 16 | 1 | 65 | 324 | 847 | 589 | 7.86E-104 |
| LibP | 5262-57 | gi|268831774|emb|HE176637.1| | 95.781 | 237 | | 10 | 0 | 1 | 237 | 430 | 666 | 2.83E-103 |
| LibP | 5262-57 | gi|80111534|emb|CT426965.1| | 95.041 | 242 | | 11 | 1 | 83 | 324 | 828 | 588 | 3.66E-102 |
| LibP | 5262-57 | gi|80060732|emb|CT403375.1| | 97.297 | 222 | | 6 | 0 | 106 | 327 | 686 | 465 | 1.32E-101 |
| LibP | 5262-57 | gi|79762271|emb|CT286022.1| | 92.748 | 262 | | 18 | 1 | 63 | 324 | 798 | 538 | 1.32E-101 |
| LibP | 5262-57 | gi|268835907|emb|HE174971.1| | 95.339 | 236 | | 11 | 0 | 1 | 236 | 430 | 665 | 4.73E-101 |
| LibP | 5262-57 | gi|79858953|emb|CT313383.1| | 96.087 | 230 | | 9 | 0 | 73 | 302 | 1 | 230 | 4.73E-101 |
| LibP | 5262-57 | gi|80043312|emb|CT395191.1| | 93.117 | 247 | | 16 | 1 | 78 | 324 | 830 | 585 | 1.32E-96 |
| LibP | 5262-57 | gi|268863452|emb|HE098624.1| | 92.43 | 251 | | 18 | 1 | 1 | 251 | 25 | 274 | 1.71E-95 |
| LibP | 5262-57 | gi|288713258|emb|HE463830.1| | 98.492 | 199 | | 3 | 0 | 1 | 199 | 423 | 621 | 7.97E-94 |
| LibP | 5262-57 | gi|268913573|emb|HE328486.1| | 91.406 | 256 | | 22 | 0 | 1 | 256 | 253 | 508 | 7.97E-94 |
| LibP | 5262-57 | gi|79719394|emb|CT230114.1| | 95.455 | 220 | | 7 | 3 | 90 | 308 | 1 | 218 | 1.03E-92 |
| LibP | 5262-57 | gi|268718520|emb|HE091270.1| | 92.245 | 245 | | 18 | 1 | 80 | 324 | 1 | 244 | 3.71E-92 |
| LibP | 5262-57 | gi|79775004|emb|CT166598.1| | 93.562 | 233 | | 14 | 1 | 92 | 324 | 1 | 232 | 3.71E-92 |
| LibP | 5262-57 | gi|79739727|emb|CT285751.1| | 93.22 | 236 | | 12 | 4 | 94 | 327 | 1 | 234 | 1.33E-91 |
| LibP | 5262-57 | gi|79702002|emb|CT293478.1| | 94.595 | 222 | | 11 | 1 | 103 | 324 | 763 | 543 | 4.80E-91 |
| LibP | 5262-57 | gi|288730395|emb|HE550849.1| | 97.949 | 195 | | 4 | 0 | 1 | 195 | 475 | 669 | 6.21E-90 |
| LibP | 5262-57 | gi|269003245|emb|HE185519.1| | 87.847 | 288 | | 33 | 2 | 1 | 287 | 398 | 684 | 2.23E-89 |
| LibP | 5262-57 | gi|79671850|emb|CT144756.1| | 92.735 | 234 | | 15 | 2 | 91 | 324 | 1 | 232 | 2.23E-89 |
| LibP | 5262-57 | gi|268864606|emb|HE105927.1| | 98.925 | 186 | | 2 | 0 | 1 | 186 | 186 | 1 | 2.89E-88 |
| LibP | 5262-57 | gi|288593755|emb|HE532907.1| | 97.382 | 191 | | 5 | 0 | 137 | 327 | 1 | 191 | 4.83E-86 |
| LibP | 5262-57 | gi|268989938|emb|HE205922.1| | 97.382 | 191 | | 5 | 0 | 137 | 327 | 1 | 191 | 4.83E-86 |
| LibP | 5262-57 | gi|79921045|emb|CT348985.1| | 92.825 | 223 | | 14 | 2 | 107 | 327 | 1 | 223 | 1.74E-85 |
| LibP | 5262-57 | gi|79905567|emb|CT343119.1| | 91.379 | 232 | | 17 | 3 | 93 | 324 | 1 | 229 | 1.05E-82 |
| LibP | 5262-57 | gi|268830801|emb|HE170119.1| | 97.268 | 183 | | 5 | 0 | 1 | 183 | 441 | 623 | 1.35E-81 |
| LibP | 5262-57 | gi|80109627|emb|CT425426.1| | 90.83 | 229 | | 20 | 1 | 96 | 324 | 1 | 228 | 6.29E-80 |
| LibP | 5262-57 | gi|269028436|emb|HE128158.1| | 84.74 | 308 | | 41 | 6 | 1 | 306 | 419 | 116 | 2.26E-79 |
| LibP | 5262-57 | gi|268912207|emb|HE329786.1| | 99.398 | 166 | | 1 | 0 | 1 | 166 | 481 | 646 | 8.14E-79 |
| LibP | 5262-57 | gi|268913261|emb|HE325090.1| | 98.81 | 168 | | 2 | 0 | 1 | 168 | 168 | 1 | 2.93E-78 |
| LibP | 5262-57 | gi|268912020|emb|HE329599.1| | 98.81 | 168 | | 2 | 0 | 1 | 168 | 168 | 1 | 2.93E-78 |
| LibP | 5262-57 | gi|268694517|emb|HE334460.1| | 98.802 | 167 | | 2 | 0 | 1 | 167 | 481 | 647 | 1.05E-77 |
| LibP | 5262-57 | gi|288770843|emb|HE400311.1| | 98.795 | 166 | | 2 | 0 | 1 | 166 | 473 | 638 | 3.79E-77 |
| LibP | 5262-57 | gi|268729419|emb|HE033339.1| | 91.284 | 218 | | 17 | 2 | 77 | 292 | 12 | 229 | 3.79E-77 |
| LibP | 5262-57 | gi|80116111|emb|CT429050.1| | 88.559 | 236 | | 26 | 1 | 89 | 324 | 1 | 235 | 1.76E-75 |
| LibP | 5262-57 | gi|79680494|emb|CT145111.1| | 93.782 | 193 | | 11 | 1 | 97 | 289 | 2 | 193 | 6.34E-75 |
| LibP | 5262-57 | gi|79872509|emb|CT326939.1| | 89.474 | 228 | | 20 | 4 | 98 | 324 | 1 | 225 | 8.20E-74 |
| LibP | 5262-57 | gi|268954112|emb|HE273556.1| | 90.995 | 211 | | 18 | 1 | 114 | 324 | 1 | 210 | 2.95E-73 |
| LibP | 5262-57 | gi|268950298|emb|HE276477.1| | 90.995 | 211 | | 18 | 1 | 114 | 324 | 1 | 210 | 2.95E-73 |
| LibP | 5262-57 | gi|79754804|emb|CT178199.1| | 91.667 | 204 | | 16 | 1 | 121 | 324 | 801 | 599 | 1.06E-72 |
| LibP | 5262-57 | gi|268996871|emb|HE198169.1| | 94.022 | 184 | | 11 | 0 | 1 | 184 | 553 | 736 | 3.82E-72 |
| LibP | 5262-57 | gi|79626220|emb|CT405418.1| | 92 | 200 | | 15 | 1 | 125 | 324 | 1 | 199 | 3.82E-72 |
| LibP | 5262-57 | gi|268928219|emb|HE307719.1| | 92.67 | 191 | | 13 | 1 | 1 | 191 | 536 | 725 | 1.77E-70 |
| LibP | 5262-57 | gi|79952486|emb|CT360426.1| | 90.431 | 209 | | 19 | 1 | 116 | 324 | 780 | 573 | 1.77E-70 |
| LibP | 5262-57 | gi|268999731|emb|HE189955.1| | 99.301 | 143 | | 1 | 0 | 185 | 327 | 1 | 143 | 4.97E-66 |
| LibP | 5262-57 | gi|79734379|emb|CT207709.1| | 87.069 | 232 | | 27 | 3 | 95 | 324 | 1 | 231 | 4.97E-66 |
| LibP | 5262-57 | gi|79864940|emb|CT319370.1| | 87.281 | 228 | | 26 | 3 | 97 | 324 | 1 | 225 | 1.79E-65 |
| LibP | 5262-57 | gi|79864940|emb|CT319370.1| | 76.86 | 121 | | 25 | 3 | 1 | 120 | 153 | 271 | 1.16E-07 |
| LibP | 5262-57 | gi|268772104|emb|HE262246.1| | 90.155 | 193 | | 18 | 1 | 132 | 324 | 1 | 192 | 2.99E-63 |
| LibP | 5262-57 | gi|268693969|emb|HE000759.1| | 81.57 | 293 | | 54 | 0 | 1 | 293 | 636 | 344 | 5.01E-61 |
| LibP | 5262-57 | gi|268867695|emb|HE096332.1| | 90.909 | 176 | | 15 | 1 | 149 | 324 | 1 | 175 | 8.38E-59 |
| LibP | 5262-57 | gi|79705185|emb|CT287468.1| | 89.13 | 184 | | 19 | 1 | 141 | 324 | 1 | 183 | 1.40E-56 |
| LibP | 5262-57 | gi|269014052|emb|HE151149.1| | 99.2 | 125 | | 1 | 0 | 1 | 125 | 125 | 1 | 5.04E-56 |
| LibP | 5262-57 | gi|268759508|emb|HE287499.1| | 92.903 | 155 | | 10 | 1 | 170 | 324 | 1 | 154 | 1.81E-55 |
| LibP | 5262-57 | gi|288702401|emb|HE486158.1| | 99.187 | 123 | | 1 | 0 | 1 | 123 | 483 | 605 | 6.52E-55 |
| LibP | 5262-57 | gi|288615500|emb|HE533508.1| | 79.299 | 314 | | 61 | 4 | 16 | 327 | 130 | 441 | 3.03E-53 |
| LibP | 5262-57 | gi|288615500|emb|HE533508.1| | 83.221 | 149 | | 21 | 4 | 1 | 147 | 465 | 611 | 3.14E-28 |
| LibP | 5262-57 | gi|268816446|emb|HE210071.1| | 89.222 | 167 | | 16 | 2 | 159 | 324 | 497 | 332 | 1.83E-50 |
| LibP | 5262-57 | gi|269015795|emb|HE160012.1| | 79.655 | 290 | | 53 | 6 | 16 | 302 | 200 | 486 | 2.36E-49 |
| LibP | 5262-57 | gi|51384621|gb|CL332653.1| | 92.199 | 141 | | 10 | 1 | 184 | 324 | 1 | 140 | 1.10E-47 |
| LibP | 5262-57 | gi|288596462|emb|HE387559.1| | 99.083 | 109 | | 1 | 0 | 1 | 109 | 109 | 1 | 3.95E-47 |
| LibP | 5262-57 | gi|268917731|emb|HE327156.1| | 100 | 100 | | 0 | 0 | 1 | 100 | 100 | 1 | 8.56E-44 |
| LibP | 5262-57 | gi|80215848|emb|CT474862.1| | 88.742 | 151 | | 16 | 1 | 174 | 324 | 593 | 444 | 3.08E-43 |
| LibP | 5262-57 | gi|268700736|emb|HE141063.1| | 98.077 | 104 | | 2 | 0 | 1 | 104 | 104 | 1 | 1.11E-42 |
| LibP | 5262-57 | gi|268835768|emb|HE173240.1| | 84.181 | 177 | | 28 | 0 | 4 | 180 | 177 | 1 | 6.66E-40 |
| LibP | 5262-57 | gi|288654687|emb|HE559572.1| | 82.796 | 186 | | 28 | 4 | 1 | 184 | 474 | 657 | 4.01E-37 |
| LibP | 5262-57 | gi|288676453|emb|HE490217.1| | 95.96 | 99 | | 4 | 0 | 1 | 99 | 519 | 617 | 1.44E-36 |
| LibP | 5262-57 | gi|268812648|emb|HE214599.1| | 88.722 | 133 | | 14 | 1 | 192 | 324 | 1 | 132 | 1.44E-36 |
| LibP | 5262-57 | gi|269058319|emb|HE040604.1| | 98.851 | 87 | | 1 | 0 | 1 | 87 | 687 | 773 | 6.71E-35 |
| LibP | 5262-57 | gi|268989062|emb|HE214143.1| | 75.399 | 313 | | 70 | 6 | 9 | 318 | 349 | 657 | 1.45E-31 |
| LibP | 5262-57 | gi|268989062|emb|HE214143.1| | 84.615 | 130 | | 16 | 4 | 6 | 133 | 445 | 572 | 5.26E-26 |
| LibP | 5262-57 | gi|268966518|emb|HE261369.1| | 87.2 | 125 | | 15 | 1 | 200 | 324 | 1 | 124 | 1.88E-30 |
| LibP | 5262-57 | gi|78585880|emb|CT103882.1| | 91.346 | 104 | | 7 | 2 | 1 | 103 | 151 | 49 | 1.88E-30 |
| LibP | 5262-57 | gi|268750357|emb|HE358253.1| | 95.349 | 86 | | 4 | 0 | 1 | 86 | 586 | 671 | 2.43E-29 |
| LibP | 5262-57 | gi|268717747|emb|HE093476.1| | 95.294 | 85 | | 4 | 0 | 1 | 85 | 85 | 1 | 8.74E-29 |
| LibP | 5262-57 | gi|269007514|emb|HE161061.1| | 83.221 | 149 | | 21 | 4 | 1 | 147 | 18 | 164 | 3.14E-28 |
| LibP | 5262-57 | gi|268716161|emb|HE090893.1| | 74.448 | 317 | | 73 | 7 | 15 | 327 | 339 | 27 | 4.07E-27 |
| LibP | 5262-57 | gi|79828922|emb|CT274737.1| | 74.448 | 317 | | 73 | 7 | 15 | 327 | 442 | 754 | 4.07E-27 |
| LibP | 5262-57 | gi|288714392|emb|HE405337.1| | 74.426 | 305 | | 70 | 7 | 27 | 327 | 662 | 362 | 1.89E-25 |
| LibP | 5262-57 | gi|288714392|emb|HE405337.1| | 74.426 | 305 | | 70 | 7 | 27 | 327 | 662 | 362 | 1.89E-25 |
| LibN | Contig2 | gi|288702401|emb|HE486158.1| | 89.313 | 262 | | 22 | 5 | 35 | 291 | 316 | 576 | 1.78E-85 |
| LibN | Contig2 | gi|269056048|emb|HE029483.1| | 89.615 | 260 | | 19 | 8 | 75 | 328 | 777 | 520 | 1.78E-85 |
| LibN | Contig2 | gi|268950875|emb|HE279831.1| | 86.469 | 303 | | 29 | 11 | 35 | 328 | 133 | 432 | 6.40E-85 |
| LibN | Contig2 | gi|268912207|emb|HE329786.1| | 86.469 | 303 | | 29 | 11 | 35 | 328 | 314 | 613 | 6.40E-85 |
| LibN | Contig2 | gi|268917731|emb|HE327156.1| | 88.931 | 262 | | 23 | 5 | 35 | 291 | 267 | 7 | 8.28E-84 |
| LibN | Contig2 | gi|288713258|emb|HE463830.1| | 86.139 | 303 | | 30 | 11 | 35 | 328 | 256 | 555 | 2.98E-83 |
| LibN | Contig2 | gi|268913261|emb|HE325090.1| | 86.139 | 303 | | 30 | 11 | 35 | 328 | 335 | 36 | 2.98E-83 |
| LibN | Contig2 | gi|268912020|emb|HE329599.1| | 86.139 | 303 | | 30 | 11 | 35 | 328 | 335 | 36 | 2.98E-83 |
| LibN | Contig2 | gi|268891780|emb|HE047306.1| | 86.139 | 303 | | 30 | 11 | 35 | 328 | 524 | 225 | 2.98E-83 |
| LibN | Contig2 | gi|268772881|emb|HE247166.1| | 86.139 | 303 | | 30 | 11 | 35 | 328 | 161 | 460 | 2.98E-83 |
| LibN | Contig2 | gi|268694517|emb|HE334460.1| | 86.139 | 303 | | 30 | 11 | 35 | 328 | 314 | 613 | 2.98E-83 |
| LibN | Contig2 | gi|288730395|emb|HE550849.1| | 88.55 | 262 | | 24 | 5 | 35 | 291 | 308 | 568 | 3.85E-82 |
| LibN | Contig2 | gi|288635963|emb|HE479338.1| | 88.55 | 262 | | 24 | 5 | 35 | 291 | 213 | 473 | 3.85E-82 |
| LibN | Contig2 | gi|288596462|emb|HE387559.1| | 88.55 | 262 | | 24 | 5 | 35 | 291 | 276 | 16 | 3.85E-82 |
| LibN | Contig2 | gi|268902279|emb|HE002591.1| | 88.55 | 262 | | 24 | 5 | 35 | 291 | 57 | 317 | 3.85E-82 |
| LibN | Contig2 | gi|79828921|emb|CT274736.1| | 88.55 | 262 | | 24 | 5 | 35 | 291 | 452 | 192 | 3.85E-82 |
| LibN | Contig2 | gi|268864606|emb|HE105927.1| | 88.55 | 262 | | 23 | 6 | 35 | 291 | 352 | 93 | 1.38E-81 |
| LibN | Contig2 | gi|269014052|emb|HE151149.1| | 88.168 | 262 | | 25 | 5 | 35 | 291 | 292 | 32 | 1.79E-80 |
| LibN | Contig2 | gi|268830801|emb|HE170119.1| | 88.168 | 262 | | 25 | 5 | 35 | 291 | 274 | 534 | 1.79E-80 |
| LibN | Contig2 | gi|268700736|emb|HE141063.1| | 88.168 | 262 | | 25 | 5 | 35 | 291 | 271 | 11 | 1.79E-80 |
| LibN | Contig2 | gi|288770843|emb|HE400311.1| | 85.479 | 303 | | 32 | 11 | 35 | 328 | 306 | 605 | 6.44E-80 |
| LibN | Contig2 | gi|269038613|emb|HE099864.1| | 85.479 | 303 | | 32 | 11 | 35 | 328 | 467 | 168 | 6.44E-80 |
| LibN | Contig2 | gi|288707504|emb|HE430474.1| | 92.166 | 217 | | 14 | 3 | 75 | 289 | 579 | 364 | 2.32E-79 |
| LibN | Contig2 | gi|288676453|emb|HE490217.1| | 88.077 | 260 | | 25 | 5 | 35 | 289 | 352 | 610 | 2.32E-79 |
| LibN | Contig2 | gi|268996871|emb|HE198169.1| | 88.077 | 260 | | 25 | 5 | 35 | 289 | 386 | 644 | 2.32E-79 |
| LibN | Contig2 | gi|268966898|emb|HE259573.1| | 88.077 | 260 | | 25 | 5 | 35 | 289 | 64 | 322 | 2.32E-79 |
| LibN | Contig2 | gi|268928219|emb|HE307719.1| | 88.077 | 260 | | 25 | 5 | 35 | 289 | 369 | 627 | 2.32E-79 |
| LibN | Contig2 | gi|268754310|emb|HE301064.1| | 88.327 | 257 | | 24 | 5 | 38 | 289 | 708 | 453 | 2.32E-79 |
| LibN | Contig2 | gi|268779272|emb|HE255902.1| | 87.786 | 262 | | 26 | 5 | 35 | 291 | 35 | 295 | 8.33E-79 |
| LibN | Contig2 | gi|269006791|emb|HE177596.1| | 87.405 | 262 | | 27 | 5 | 35 | 291 | 575 | 315 | 3.88E-77 |
| LibN | Contig2 | gi|269058319|emb|HE040604.1| | 87.843 | 255 | | 25 | 5 | 35 | 284 | 520 | 773 | 1.39E-76 |
| LibN | Contig2 | gi|269041112|emb|HE085726.1| | 91.364 | 220 | | 11 | 8 | 115 | 328 | 644 | 427 | 1.39E-76 |
| LibN | Contig2 | gi|288614170|emb|HE548276.1| | 90.868 | 219 | | 18 | 2 | 75 | 291 | 396 | 178 | 5.02E-76 |
| LibN | Contig2 | gi|269036928|emb|HE092449.1| | 90.909 | 220 | | 16 | 4 | 75 | 291 | 489 | 271 | 5.02E-76 |
| LibN | Contig2 | gi|268841517|emb|HE159056.1| | 87.398 | 246 | | 24 | 6 | 35 | 274 | 351 | 595 | 5.05E-71 |
| LibN | Contig2 | gi|288615500|emb|HE533508.1| | 81.073 | 317 | | 52 | 7 | 23 | 332 | 286 | 601 | 3.96E-62 |
| LibN | Contig2 | gi|269063684|emb|HE008252.1| | 86.207 | 232 | | 25 | 6 | 35 | 261 | 594 | 823 | 1.42E-61 |
| LibN | Contig2 | gi|268765006|emb|HE272786.1| | 91.257 | 183 | | 10 | 6 | 150 | 328 | 505 | 325 | 1.42E-61 |
| LibN | Contig2 | gi|288626051|emb|HE493425.1| | 80.878 | 319 | | 52 | 7 | 1 | 313 | 353 | 38 | 5.12E-61 |
| LibN | Contig2 | gi|288614209|emb|HE549300.1| | 81.23 | 309 | | 51 | 6 | 1 | 303 | 59 | 366 | 5.12E-61 |
| LibN | Contig2 | gi|288748491|emb|HE496608.1| | 80.564 | 319 | | 52 | 8 | 1 | 313 | 329 | 15 | 2.38E-59 |
| LibN | Contig2 | gi|288729374|emb|HE525833.1| | 80.906 | 309 | | 52 | 6 | 1 | 303 | 174 | 481 | 2.38E-59 |
| LibN | Contig2 | gi|288664595|emb|HE539714.1| | 80.564 | 319 | | 53 | 7 | 1 | 313 | 475 | 160 | 2.38E-59 |
| LibN | Contig2 | gi|269067132|emb|HE040904.1| | 80.564 | 319 | | 53 | 7 | 1 | 313 | 337 | 22 | 2.38E-59 |
| LibN | Contig2 | gi|269050142|emb|HE053071.1| | 80.906 | 309 | | 52 | 6 | 1 | 303 | 273 | 580 | 2.38E-59 |
| LibN | Contig2 | gi|268896015|emb|HE018617.1| | 80.906 | 309 | | 52 | 6 | 1 | 303 | 163 | 470 | 2.38E-59 |
| LibN | Contig2 | gi|268895999|emb|HE018601.1| | 80.906 | 309 | | 52 | 6 | 1 | 303 | 163 | 470 | 2.38E-59 |
| LibN | Contig2 | gi|268746443|emb|HE351963.1| | 80.564 | 319 | | 53 | 7 | 1 | 313 | 449 | 134 | 2.38E-59 |
| LibN | Contig2 | gi|268701259|emb|HE114444.1| | 80.564 | 319 | | 53 | 7 | 1 | 313 | 420 | 105 | 2.38E-59 |
| LibN | Contig2 | gi|268913099|emb|HE328892.1| | 80.844 | 308 | | 52 | 6 | 1 | 302 | 332 | 26 | 8.57E-59 |
| LibN | Contig2 | gi|288753247|emb|HE470154.1| | 80.583 | 309 | | 53 | 6 | 1 | 303 | 47 | 354 | 1.11E-57 |
| LibN | Contig2 | gi|79939385|emb|CT355906.1| | 79.758 | 331 | | 58 | 8 | 11 | 334 | 357 | 685 | 1.11E-57 |
| LibN | Contig2 | gi|79939385|emb|CT355906.1| | 77.876 | 113 | | 19 | 6 | 225 | 334 | 80 | 189 | 1.19E-07 |
| LibN | Contig2 | gi|268693205|emb|HE021494.1| | 81.081 | 296 | | 49 | 6 | 1 | 290 | 160 | 454 | 3.99E-57 |
| LibN | Contig2 | gi|268730814|emb|HE048881.1| | 79.937 | 319 | | 55 | 7 | 1 | 313 | 339 | 24 | 5.16E-56 |
| LibN | Contig2 | gi|268867420|emb|HE093877.1| | 81.119 | 286 | | 47 | 6 | 1 | 281 | 110 | 393 | 6.68E-55 |
| LibN | Contig2 | gi|288727933|emb|HE491057.1| | 80.065 | 306 | | 54 | 6 | 4 | 303 | 1 | 305 | 2.40E-54 |
| LibN | Contig2 | gi|269056191|emb|HE029626.1| | 79.154 | 331 | | 60 | 8 | 11 | 334 | 196 | 524 | 2.40E-54 |
| LibN | Contig2 | gi|269004063|emb|HE164352.1| | 79.935 | 309 | | 55 | 6 | 1 | 303 | 174 | 481 | 2.40E-54 |
| LibN | Contig2 | gi|268762198|emb|HE285827.1| | 79.624 | 319 | | 56 | 7 | 1 | 313 | 409 | 94 | 2.40E-54 |
| LibN | Contig2 | gi|268868195|emb|HE094257.1| | 90.854 | 164 | | 13 | 2 | 75 | 236 | 164 | 1 | 8.63E-54 |
| LibN | Contig2 | gi|79801853|emb|CT284366.1| | 81.923 | 260 | | 42 | 4 | 1 | 256 | 559 | 817 | 1.12E-52 |
| LibN | Contig2 | gi|268952882|emb|HE286600.1| | 81.712 | 257 | | 42 | 4 | 1 | 253 | 256 | 1 | 5.20E-51 |
| LibN | Contig2 | gi|268887004|emb|HE031656.1| | 78.55 | 331 | | 62 | 8 | 11 | 334 | 477 | 149 | 5.20E-51 |
| LibN | Contig2 | gi|268856413|emb|HE110406.1| | 79.1 | 311 | | 56 | 7 | 10 | 314 | 608 | 301 | 1.87E-50 |
| LibN | Contig2 | gi|269013633|emb|HE145835.1| | 82.7 | 237 | | 36 | 4 | 1 | 233 | 128 | 363 | 6.72E-50 |
| LibN | Contig2 | gi|79626223|emb|CT405419.1| | 78.248 | 331 | | 65 | 7 | 10 | 334 | 616 | 287 | 6.72E-50 |
| LibN | Contig2 | gi|79626223|emb|CT405419.1| | 84.264 | 197 | | 22 | 8 | 35 | 225 | 194 | 1 | 3.15E-43 |
| LibN | Contig2 | gi|78593383|emb|CT108913.1| | 78.248 | 331 | | 65 | 7 | 10 | 334 | 591 | 262 | 6.72E-50 |
| LibN | Contig2 | gi|78585880|emb|CT103882.1| | 79.288 | 309 | | 53 | 10 | 34 | 334 | 318 | 13 | 6.72E-50 |
| LibN | Contig2 | gi|288761486|emb|HE470475.1| | 79.725 | 291 | | 52 | 6 | 19 | 303 | 1 | 290 | 2.42E-49 |
| LibN | Contig2 | gi|268754306|emb|HE301060.1| | 90 | 160 | | 10 | 6 | 173 | 328 | 619 | 462 | 2.42E-49 |
| LibN | Contig2 | gi|288583948|emb|HE557603.1| | 82.759 | 232 | | 35 | 4 | 1 | 228 | 231 | 1 | 8.70E-49 |
| LibN | Contig2 | gi|268835907|emb|HE174971.1| | 80.22 | 273 | | 48 | 5 | 23 | 291 | 253 | 523 | 3.13E-48 |
| LibN | Contig2 | gi|268831774|emb|HE176637.1| | 80.22 | 273 | | 48 | 5 | 23 | 291 | 253 | 523 | 3.13E-48 |
| LibN | Contig2 | gi|268835768|emb|HE173240.1| | 83.486 | 218 | | 30 | 6 | 67 | 280 | 263 | 48 | 1.12E-47 |
| LibN | Contig2 | gi|80044650|emb|CT395722.1| | 84.135 | 208 | | 28 | 4 | 1 | 204 | 217 | 11 | 4.05E-47 |
| LibN | Contig2 | gi|79785620|emb|CT245088.1| | 84.135 | 208 | | 28 | 4 | 1 | 204 | 217 | 11 | 4.05E-47 |
| LibN | Contig2 | gi|268726440|emb|HE350572.1| | 85.052 | 194 | | 25 | 3 | 1 | 190 | 196 | 3 | 1.46E-46 |
| LibN | Contig2 | gi|78593328|emb|CT108858.1| | 82.667 | 225 | | 33 | 5 | 1 | 221 | 223 | 1 | 1.46E-46 |
| LibN | Contig2 | gi|288699918|emb|HE390832.1| | 77.644 | 331 | | 65 | 8 | 11 | 334 | 69 | 397 | 5.23E-46 |
| LibN | Contig2 | gi|288748882|emb|HE506741.1| | 84.896 | 192 | | 25 | 3 | 1 | 188 | 192 | 1 | 1.88E-45 |
| LibN | Contig2 | gi|288666959|emb|HE555108.1| | 81.897 | 232 | | 37 | 4 | 1 | 228 | 231 | 1 | 1.88E-45 |
| LibN | Contig2 | gi|269013220|emb|HE164974.1| | 81.938 | 227 | | 39 | 2 | 67 | 291 | 286 | 60 | 1.88E-45 |
| LibN | Contig2 | gi|269003245|emb|HE185519.1| | 79.63 | 270 | | 51 | 4 | 26 | 291 | 222 | 491 | 1.88E-45 |
| LibN | Contig2 | gi|268966372|emb|HE246375.1| | 79.705 | 271 | | 50 | 4 | 68 | 334 | 1 | 270 | 1.88E-45 |
| LibN | Contig2 | gi|268852741|emb|HE142199.1| | 81.938 | 227 | | 39 | 2 | 67 | 291 | 286 | 60 | 1.88E-45 |
| LibN | Contig2 | gi|268750357|emb|HE358253.1| | 80 | 265 | | 47 | 5 | 23 | 283 | 409 | 671 | 1.88E-45 |
| LibN | Contig2 | gi|268693969|emb|HE000759.1| | 86.441 | 177 | | 20 | 4 | 115 | 288 | 721 | 546 | 1.88E-45 |
| LibN | Contig2 | gi|80088118|emb|CT415550.1| | 82.222 | 225 | | 34 | 5 | 1 | 221 | 223 | 1 | 6.77E-45 |
| LibN | Contig2 | gi|79867880|emb|CT322310.1| | 82.222 | 225 | | 34 | 5 | 1 | 221 | 223 | 1 | 6.77E-45 |
| LibN | Contig2 | gi|268863452|emb|HE098624.1| | 95.69 | 116 | | 5 | 0 | 174 | 289 | 1 | 116 | 2.44E-44 |
| LibN | Contig2 | gi|78656048|emb|CT102641.1| | 83.173 | 208 | | 29 | 5 | 1 | 204 | 208 | 3 | 8.76E-44 |
| LibN | Contig2 | gi|269067650|emb|HE043222.1| | 84.946 | 186 | | 22 | 5 | 35 | 215 | 484 | 668 | 3.15E-43 |
| LibN | Contig2 | gi|269013840|emb|HE147434.1| | 85.475 | 179 | | 22 | 3 | 1 | 175 | 179 | 1 | 3.15E-43 |
| LibN | Contig2 | gi|268717747|emb|HE093476.1| | 79.545 | 264 | | 48 | 5 | 23 | 282 | 262 | 1 | 3.15E-43 |
| LibN | Contig2 | gi|268778170|emb|HE255800.1| | 79.842 | 253 | | 46 | 5 | 8 | 256 | 266 | 15 | 4.07E-42 |
| LibN | Contig2 | gi|78673658|emb|CT137675.1| | 82.692 | 208 | | 31 | 4 | 1 | 204 | 211 | 5 | 4.07E-42 |
| LibN | Contig2 | gi|288702252|emb|HE481235.1| | 86.585 | 164 | | 18 | 3 | 1 | 160 | 406 | 569 | 1.47E-41 |
| LibN | Contig2 | gi|79781513|emb|CT303184.1| | 81.25 | 224 | | 37 | 4 | 1 | 221 | 222 | 1 | 5.27E-41 |
| LibN | Contig2 | gi|268940813|emb|HE293346.1| | 77.891 | 294 | | 56 | 7 | 26 | 313 | 453 | 163 | 1.90E-40 |
| LibN | Contig2 | gi|80160197|emb|CT448896.1| | 78.169 | 284 | | 54 | 8 | 10 | 287 | 95 | 376 | 1.90E-40 |

^1^ Sequence accession number (GenBank/GSS name) of the matched sequence.

^2^ Percentage of identity of the aligned region.

^3^ Query sequence length.

^4^ Number of mismatches in the alignment.

^5^ Number of gaps in the alignment.

^6^ Start of alignment in query.

^7^ End of alignment in query.

^8^ Start of alignment in subject.

^9^ End of alignment in subject.

^10^ Expected value of the alignment.
